# Altered functional brain organisation in preterm Children: Motor task and resting-state fMRI findings at six years

**DOI:** 10.1016/j.nicl.2025.103906

**Published:** 2025-11-08

**Authors:** Javier Urriola, Kerstin Pannek, DanaKai Bradford, Jurgen Fripp, Julie Trinder, Karen Mistry, Paul B Colditz, Roslyn Boyd, Samudragupta Bora, Joanne M. George, Alex M. Pagnozzi

**Affiliations:** a Australian e-Health Research Centre, CSIRO, Brisbane, Australia; b School of Electrical Engineering and Computer Science, Faculty of Engineering, Architecture and Information Technology, The University of Queensland, Brisbane, Australia; c Child Health Research Centre, Faculty of Health, Medicine and Behavioural Sciences, The University of Queensland, Brisbane, Australia; d Perinatal Research Centre, University of Queensland Centre for Clinical Research (UQCCR), Brisbane, Australia; e Queensland Cerebral Palsy and Rehabilitation Research Centre, Faculty of Medicine, The University of Queensland, Brisbane, Australia; f Mater Research Institute, Faculty of Health, Medicine and Behavioural Sciences, The University of Queensland, Brisbane, Australia; g Health Services Research Center, University Hospitals Research & Education Institute Department of Pediatrics, University Hospitals Rainbow Babies & Children’s Hospital, Case Western Reserve University School of Medicine, Cleveland, OH, USA; h Physiotherapy Department, Queensland Children’s Hospital, Children’s Health Queensland Hospital and Health Service, Brisbane, Australia

**Keywords:** Preterm children, Motor network, Task-based fMRI, Resting-state fMRI, Functional connectivity

## Abstract

Very preterm birth significantly increases the risk of lifelong cognitive and motor deficits by disrupting early brain development. We characterised functional brain organisation at six years of age in children born very preterm (VPT) compared to term-born controls (TC). Functional MRI comprised i) visually cued ∼1 Hz right- and left-hand tapping analysed with a general linear model and permutation testing, and ii) eyes-closed resting-state acquisitions assessed with ROI-to-ROI connectivity for cortical/cerebellar and subcortical networks.

Seventy-one children born <31 weeks gestation were scanned; high-quality task data were available from 58 (43 VPT, 15 TC) for right-hand and 48 (36 VPT, 12 TC) for left-hand tapping, and resting-state data from 40 (28 VPT, 12 TC). Both groups activated expected motor regions, but VPT children showed greater activation and stronger left-temporal lateralisation during right-hand tapping (p < 0.032). Resting-state analyses revealed weaker connectivity in VPT children within striatal circuits and between salience, dorsal-attention, and visual networks, alongside reduced anticorrelation between default-mode and frontoparietal networks.

Our findings indicate that children born very preterm exhibit a persistent reorganisation of both task-evoked motor activation and large-scale cortical and subcortical resting-state network connectivity at six years of age. These differences in functional brain organisation provide insights into long-lasting neurobiological changes associated with very preterm birth during a critical period of child development.

## Highlights

• VPT children have increased right-hand motor response and lateralisation.
• Connectivity between task-positive networks is reduced in VPT children.
• Anticorrelation between DMN and FPN is decreased in VPT children.
• Subcortical connectivity is notably weaker in VPT children.

## 1 Introduction

Preterm birth is a major risk factor for adverse neurodevelopmental outcomes in children. These outcomes can range from motor to higher-order cognitive deficits, which may persist throughout childhood and into adulthood (Anderson et al., 2021, Cheong et al., 2017, Cheong et al., 2024, Hee Chung et al., 2020). The vulnerability to these long-term adverse outcomes is particularly pronounced in very preterm (VPT) children, i.e. those born before 32 weeks of gestation (Fawke, 2007). Research using magnetic resonance imaging (MRI) has revealed that VPT birth is associated with a variety of region-specific structural abnormalities. These include a reduction of cortical grey matter volume in sensorimotor, frontal and temporal cortices, alongside reduced cortical folding in the lateral temporal cortex (Zhang et al., 2015). Volumetric reductions also extend to subcortical deep grey matter structures, such as the thalamus and striatum (Meng et al., 2016); all of which are known to be crucial for motor and cognitive functions and have long-term effects that persist into adolescence (Kelly et al., 2023) and adulthood (Allin et al., 2011, Eikenes et al., 2011, Nosarti et al., 2014).

Motor impairments, including cerebral palsy and neuromotor dysfunction, are common in VPT children, significantly impacting their daily functioning and quality of life (Edwards et al., 2011, Spittle and Orton, 2014). While much research has focused on clinical outcomes and brain structural differences, understanding the functional neurobiological mechanisms underlying these developmental deficits is key for improving detection and intervention strategies in this high-risk population.

Functional magnetic resonance imaging (fMRI) has been used to reveal altered brain function in individuals born VPT, with task-based approaches used to isolate and identify neural substrates of specific deficits. During hand-tapping motor tasks, VPT adolescents and adults have exhibited differential brain activation compared to term-born controls, often involving increased brain activation, despite comparable task performance (Lawrence et al., 2014, Uusitalo et al., 2021). This pattern, interpreted as compensatory reorganisation or reduced processing efficiency, persists from adolescence into adulthood. By contrast, data from early school-age VPT children are scarce, despite this stage combining rapidly increasing environmental motor demands with a high capacity for experience-dependent neuroplastic change (Weyandt et al., 2020).

Resting-state fMRI complements task studies by mapping intrinsic large-scale network organisation (Fox and Raichle, 2007). In preterm cohorts, widespread disruptions in functional connectivity (FC) have been reported across subcortical and cortical circuits (Cho et al., 2022, Wehrle et al., 2018, Wheelock et al., 2018). These resting-state functional connectivity (rs-FC) alterations are linked to neurodevelopmental outcomes (Cho et al., 2022, Rogers et al., 2018, Smyser et al., 2016, Wehrle et al., 2018, Wheelock et al., 2018, Wheelock et al., 2021) and mirror known structural vulnerabilities (Abernethy et al., 2004, Meng et al., 2016, Nosarti et al., 2008, Nosarti et al., 2014, Peterson et al., 2000, Srinivasan et al., 2007).

To our knowledge, this study is novel in examining both motor task-based and resting-state fMRI measures in a cohort of VPT children at early school age, when functional impairments often become more apparent. This dual fMRI approach provides a more comprehensive view by identifying atypical neural recruitment during specific motor processes through task-based fMRI and revealing the intrinsic functional architecture across the entire brain with resting-state fMRI. Consistent with recent work showing state-dependent differences in large-scale networks in school-aged children born extremely preterm across rest and cognitive task states (Tokariev et al., 2025), we examined both task-evoked responses and resting-state connectivity. Building on these precedents, we collected fMRI data from six-year-old VPT children and term-born controls, including: task-based scans during a visually cued sequential hand-tapping task (performed with each hand separately), and eyes-closed rs-fMRI. We tested two primary hypotheses: (1) Compared with term-born controls, VPT children would show greater and/or more widespread BOLD activation while performing the visually-cued hand-tapping task, and (2) VPT children would exhibit widespread alterations in functional connectivity, particularly weaker connectivity within subcortical circuits and altered coupling between task-positive and task-negative resting-state networks relative to their term-born peers. By integrating complementary fMRI modalities in a well-characterised early-school age cohort, we aim to provide a more comprehensive account of post-preterm functional brain organisation and to identify candidate neural targets for early interventions.

## 2 Methods

### 2.1 Participants and inclusion criteria

Participants were recruited from the Prediction of Preterm Brain Outcomes at 6 years (PREBO-6) prospective study (George et al., 2020), with MRI scans conducted between 2019 and 2023. These children were originally enrolled as infants in the Prediction of Preterm Motor Outcomes (PPREMO)(George et al., 2015) or the Preterm Brain Outcomes (PREBO) studies, which recruited infants born at less than 31 weeks’ gestation at the Royal Brisbane and Women's Hospital (RBWH) in Brisbane, Australia, between January 2013 and December 2019. Additional inclusion criteria for the PPREMO and PREBO studies were English-speaking families living within 200 km of RBWH, and no congenital or chromosomal abnormalities likely to impact neurodevelopment. A comparison group of term-born infants was also recruited during the initial PPREMO/PREBO studies. These infants were eligible if they were born between 38- and 41 weeks postmenstrual age following an uncomplicated pregnancy and delivery, had a birth weight above the 10th percentile, and were not admitted to neonatal intensive or special care units. MRI scans for the present study were conducted between 2019 and 2022, when the children were six years of age. For the 6-year follow-up PREBO-6 study, children were eligible if they were turning six years of age within the study period (2019–2022) and continued to meet the original inclusion criteria.

The PREBO-6 study received ethical approval from the institutional review board (HREC/19/QCHQ/49800) at Children's Health Queensland, with reciprocal approvals granted by The University of Queensland (2019000426) and CSIRO (2019_013_RR). Written consent was obtained from the parents/caregivers of all participating children. Trial registration ANZTR12619000155190.

To increase the pool of term controls for resting-state fMRI analysis, we extracted an age-matched term control subset from the Autism Brain Imaging Data Exchange II (ABIDE-II) (Di Martino et al., 2017) and Healthy Brain Network (HBN) (Alexander et al., 2017). Before any pooling, we prespecified a comparability analysis: ROI‑to‑ROI resting‑state connectivity would be compared between PREBO‑6 term controls (n = 12) and external term controls (n = 40) using the same GLM as the main analyses, with corrected age at MRI, sex at birth, handedness, mean framewise displacement (FD, mm), and acquisition site as covariates. Inference used non‑parametric cluster‑mass testing. If no between‑dataset differences survived correction, we would proceed with pooled resting‑state analyses, explicitly labelled ‘PREBO‑6+’; otherwise, we would report ‘PREBO‑6′ only. Full details of the external cohorts (source databases, inclusion criteria, and acquisition parameters) are provided in the Supplementary Material.

### 2.2 Data acquisition

Preparatory procedures, including mock scanning sessions and motion minimisation during the scan, are detailed in the Supplementary Material.

#### 2.2.1 Anatomical imaging

Structural, task-based fMRI and resting-state fMRI scans were performed using a 3.0 Tesla Siemens Prisma scanner (Erlangen, Germany) with a 64-channel receive-only head coil at the Herston Imaging Research Facility (HIRF) in Brisbane, Australia. A high-resolution structural magnetisation-prepared rapid acquisition gradient-echo T1-weighted (MPRAGE) image was acquired before functional time-series acquisition. The MPRAGE image had the following parameters: TR 2500 ms, TE 2.9 ms, TI 1070 ms, matrix 256 × 256, 160 slices, flip angle 8°, and effective 1 mm isotropic voxel resolution. The full acquisition protocol, which included additional imaging sequences, is described elsewhere (George et al., 2020).

#### 2.2.2 Task-Based fMRI acquisition

The task-based fMRI acquisition consisted of two T2*-weighted gradient echo planar imaging (GRE-EPI) runs. Each run had the following parameters: TR = 3 s, TE = 26 ms, flip angle = 76°, 2.6 × 2.6 mm in-plane resolution, 60 slices with 2.6 mm thickness were acquired in an interleaved order, GRAPPA acceleration factor = 2, 72 × 72 matrix, and a total acquisition time of 5:38 min. To correct for susceptibility-induced distortions, a pair of spin-echo (SE) EPI images with reversed phase-encoding directions (AP/PA) were acquired immediately prior to the task runs. These scans were geometrically matched to the task-based functional. Distortion fields were estimated from these “blip-up/blip-down” images and applied to correct the GRE-EPI data. For each task-based fMRI run, a block design motor paradigm consisting of four ON-OFF acquisitions with a 36-second duration per block was performed. Participants engaged in a hand-tapping task, adhering to a rhythm of approximately 1 Hz when prompted visually with “Move” during the “ON” period and instructed to rest with a “Stop” prompt during the “OFF” period. A researcher (KM) was present in the scanner room, observing the hand-tapping activity, while a researcher (JT) in the console room observed the EPI volume progression and the real-time blood-oxygen-level-dependent (BOLD) activation to verify task compliance. During the first fMRI run, the dominant hand was used. The procedure was repeated for the participant's non-dominant hand during the second fMRI run.

#### 2.2.3 Resting-State fMRI acquisition

The resting-state fMRI acquisition consisted of one T2*-weighted Multiband GRE-EPI run with the following parameters: TR = 0.97 s, TE = 30 ms, flip angle = 52°, 2 × 2 mm in-plane resolution, 72x2mm-thick slices acquired in intercalated order, multiband acceleration factor = 6, partial Fourier 7/8, 104 × 104 matrix, and a total acquisition ranging from 5:28 min to 6:39 min, to extract stable brain networks in school-age children (T. White et al., 2014). Susceptibility distortion correction was performed using a geometrically matched pair of SE-EPI scans with reversed phase-encoding directions. These distortion correction scans were acquired without parallel imaging acceleration (TR/TE = 6850/51 ms, echo spacing = 0.56 ms, and bandwidth = 2290 Hz/Px). During the resting-state fMRI run, participants were instructed to lie still and remain still, keeping their eyes closed and not engaging in any specific task. Compliance was monitored directly by a researcher in the scanner room (KM), while another researcher in the console room tracked the real-time EPI volume progression (JT).

### 2.3 Functional MRI analysis

#### 2.3.1 Task-Based fMRI analysis

We applied standard preprocessing (Poldrack et al., 2011) to the task-based fMRI data using FSL (FMRIB Software Library, version 6.0.5) and SPM12 (Wellcome Trust Centre for Neuroimaging, London, UK), including susceptibility-induced distortion correction, slice-timing correction, realignment, normalisation to MNI space, and spatial smoothing (6 mm Full-Width Half Maximum (FWHM)). Motion artefacts were minimised by including only participants with mean relative motion <1.3 mm and censoring volumes with framewise displacement >0.9 mm (Power et al., 2012, Siegel et al., 2014). First-level general linear models (GLMs) included task regressors convolved with the canonical hemodynamic response function and nuisance regressors for motion parameters, white matter, and CSF signals.

Group-level contrasts between the VPT and term-born control cohorts were assessed with non-parametric permutation testing in SnPM13 (https://nisox.org/Software/SnPM13; 5,000 permutations), controlling the family-wise error rate at p < 0.05. Lateralisation of the BOLD response was quantified using the Laterality Index (LI) defined as LI=(nL-nR)/(nL + nR) (Wilke and Schmithorst, 2006), with values > 0.10 indicating left lateralisation, <-0.10 indicating right lateralisation, and values between indicating bilateral activation.

#### 2.3.2 Resting-State fMRI analysis

Resting-state data underwent similar preprocessing steps, with additional denoising procedures implemented in CONN toolbox v.21a (Whitfield-Gabrieli and Nieto-Castanon, 2012). Participants with mean relative displacement >0.55 mm were excluded (Satterthwaite et al., 2013). Noise reduction included CompCor physiological noise correction (Behzadi et al., 2007), censoring of high-motion volumes (framewise displacement >0.9 mm), bandpass filtering (0.008–0.09 Hz), and regression of motion parameters and their derivatives.

For network analyses, we conducted two independent ROI-to-ROI FC investigations. This separation was hypothesis-driven, motivated by extensive evidence that preterm birth disproportionately affects the structure and function of basal ganglia-thalamic circuits (Abernethy et al., 2004, Loh et al., 2017, Meng et al., 2016, Nosarti et al., 2008, Nosarti et al., 2014, Peterson et al., 2000, Srinivasan et al., 2007), which possess a distinct underlying interconnected system (Lanciego et al., 2012, McHaffie et al., 2005), allowing for a targeted characterisation of potential within-system disruptions.

The first analysis assessed connectivity within large-scale cortical and cerebellar networks using 32 ROIs representing eight canonical resting-state networks (Default Mode, Sensorimotor, Visual, Salience, Dorsal Attention, Frontoparietal, Language, and Cerebellar) sourced from CONN’s ‘networks.nii’ file (see Supplementary Fig. S4). The second, separate analysis focused on intrinsic connectivity among 10 deep-grey matter ROIs (bilateral caudate, putamen, pallidum, nucleus accumbens, and thalamus) selected from the FSL Harvard-Oxford subcortical atlas parcellations within CONN’s ‘atlas.nii’ file (see Supplementary Fig. S5).

For both analyses, functional connectivity was quantified by first extracting the mean BOLD time-series from each pre-defined ROI for each participant. The connectivity between each pair of ROIs was then calculated as the Pearson's bivariate correlation coefficient between their respective time-series. Finally, these correlation coefficients were Fisher's z-transformed to improve normality for subsequent group-level statistical analyses. Group differences were assessed using GLMs controlling for age, sex, handedness, specific acquisition site (e.g., PREBO-6 scanner, ABIDE-NYU scanner, ABIDE-KKI scanner, HBN-CBIC scanner), and motion (mean framewise displacement), with non-parametric statistics (1,000 permutations) and cluster-level inference (voxel threshold p < 0.01, cluster-level FDR p < 0.05). Full methodological details are provided in the Supplementary Material.

### 2.4 Statistics

All statistical analyses for demographic comparisons and primary imaging data (e.g., group-level fMRI analyses) were conducted by JU using MATLAB R2021b (The MathWorks Inc, Natick, MA, USA) with SPM12 and CONN toolbox v.21a (MIT, Cambridge, MA, USA). A threshold of p < 0.05, corrected where appropriate and two-tailed, defined statistical significance. Normality of data distributions was assessed using the Shapiro-Wilk test; for non-normally distributed variables, non-parametric methods (Mann-Whitney *U* test for continuous, Fisher’s exact test for categorical) were employed. Demographic factors such as gestational age at birth (in days) and birthweight (in grams) were included. Specific analyses of partial correlations between functional connectivity values and these demographic factors, controlling for age at scan and sex at birth, were conducted by AP using R version 4.0.2 (R Core Team, 2021), with figures generated via the “ggpubr” package (Kassambara, 2018). Across all relevant analyses, multiple comparison corrections were applied using the Benjamini-Hochberg procedure, with a false discovery rate (FDR) of 5 %. For all fMRI-based group comparisons, non-parametric permutation testing was employed for statistical inference, which is robust to variations in sample size and non-normal data distributions.

## 3 Results

### 3.1 Demographic, neonatal, and behavioural data

A total of n = 122 preterm and n = 32 term children were eligible between 2019 and July 2022, of whom n = 80 preterm and n = 25 term children participated in the PREBO-6 study. Of these, 71 participants underwent MRI acquisitions (52 VPT, 19 TC), with functional MRI acquired in 66. Of these participants, 65 performed right-hand motor tasks (48 VPT; 17 TC), 61 performed left-hand motor tasks (44 VPT; 17 TC), and 60 performed both motor tasks (43 VPT; 17 TC). Resting-state fMRI data were available for 53 participants (39 VPT; 14 TC). Following data pre-processing, 58 participants passed quality control (89 % of those who performed the task) for the right-hand motor task (43 VPT; 15 TC), 48 participants (79 % of those performing the task) for the left-hand motor task (36 VPT; 12 TC) and 46 (87 %) for both tasks (35 VPT; 11 TC). For the resting-state fMRI analysis, data from 40 participants (75 %) met the quality criteria for motion (28 VPT; 12 TC). Statistical analyses revealed no significant differences between the included and excluded groups in terms of sex distribution (p > 0.99, Fisher’s exact test), age (p = 0.45, Mann-Whitney *U* test), or handedness (p = 0.46, Fisher’s exact test). A flowchart detailing data availability and loss is provided in Supplementary Material. The VPT group had a median gestational age at birth of 28 weeks (interquartile range [IQR]: 27–29 weeks) and a median birth weight of 1104 g (IQR: 803–1405 g). Term-born participants had a median gestational age of 39 weeks (IQR: 38.25–40 weeks) and a median birth weighed of 3051 g (IQR: 3385 – 3573 g). For the right-hand motor task, the groups were well-matched for chronological age at MRI, sex distribution, and handedness. A significant difference was observed in corrected age at MRI, with the VPT group being scanned at a median of 6.1 years compared to 6.3 years for the TC group (p = 0.036). For the left-hand motor task, no significant demographic differences were observed between the groups. Motion metrics did not differ between groups for either task. Full demographic details are presented in Table 1.

**Table 1:** Demographic information, perinatal data, and motion metrics for very preterm and term control groups across both task-based fMRI acquisitions.

	Participants that passed QC for the Right-hand motor task (n = 58)			Participants that passed QC for the Left-hand motor task (n = 48)		
	VPT	TC	Statistic (P)	VPT	TC	Statistic (P)
	n = 43	n = 15		n = 36	n = 12	
**Perinatal data**						
Median gestational age in weeks (IQR)	28 (27–29)	39 (38–40)	*** <2.2e-16	29 (28–29)	39 (38–40)	*** <2.2e-16
Median birth weight in grams (IQR)	1104 (370)	3512 (172)	*** <2.2e-16	1136 (429)	3501 (157)	*** <2.2e-16

**Demographic**						
Sex at birth, n (% male)	24 (55.8 %)	6 (40.0 %)	*0.37*	19 (52.8 %)	5 (41.7 %)	*0.74*
Age at MRI (years) (median, IQR)	6.3 (6.2–6.4)	6.3 (6.1–6.6)	*0.82*	6.3 (6.2–6.4)	6.5 (6 – 6.8)	*0.63*
Corrected age at MRI (years) (median, IQR)	6.1 (6.0–6.2)	6.3 (6.1–6.6)	* 0.036	6.1 (6–6.2)	6.5 (6 – 6.8)	*0.06*
Handedness (Right/Left)	(39/4)	(13/2)	*0.64*	(31/5)	(10/2)	*1*

**Motion metrics**						
RMS relative displacement (mm)	0.2 (0.1–0.4)	0.2 (0.1–0.3)	*0.6*	0.2 (0.1–0.4)	0.2 (0.1–0.5)	*0.93*
FD (mm)	0.3 (0.1–0.8)	0.3 (0.2–0.5)	*0.6*	0.3 (0.2–0.6)	0.3 (0.1–0.7)	*0.90*

Acronyms: VPT-very preterm, TC-term control, RMS-Root Mean Square, FD-Framewise Displacement Statistical significance is denoted by (*) p < 0.05, Mann-Whitney test; (**) p < 0.01; (***) p < 0.001.

### 3.2 Task-Based fMRI results

#### 3.2.1 Single subject activations

Most participants (84 %) demonstrated functional brain activation during the right and left hand-motor tasks. Specifically, for the right-hand motor tapping paradigm, 90 % (43/48) of VPT and 88 % (15/17) of TC exhibited brain activations. For the left-hand motor tapping paradigm, 82 % (36/44) of VPT participants and 71 % (12/17) of TC showed activation. We found no significant difference in the number of subjects with fMRI motor task activation between the VPT and TC groups for either hand task (p > 0.9, Chi-square test).

#### 3.2.2 Within-Group fMRI activation

**Fig. 1:**
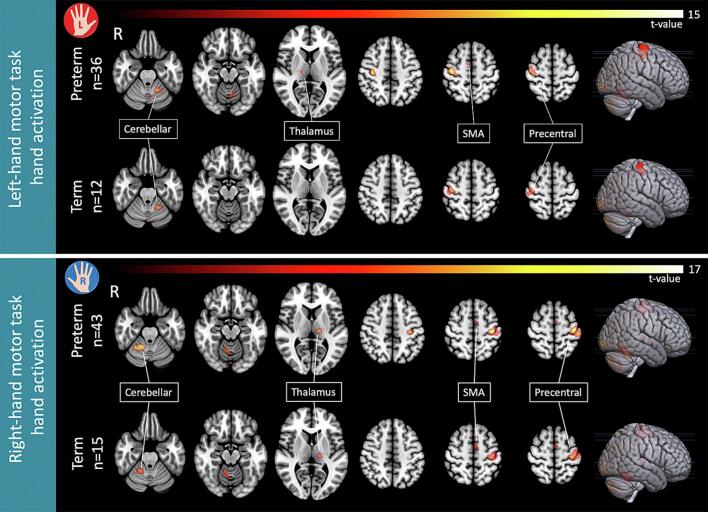
BOLD activation maps during motor tasks derived from within-group second-level analysis (p < 0.05, FWE-corrected, permutation test). Top panel: Left-hand motor task activation (Contrast: [Left hand tapping > Rest]): Preterm (n = 36, top) and Term (n = 12, bottom). Bottom panel: Right-hand motor task activation (Contrast: [Right hand tapping > Rest]): Preterm (n = 43, top) and Term (n = 15, bottom).

During the ‘Move’ condition, when contrasted to ‘Stop’, preterm and control participants showed a significant BOLD response (One-sample *t*-test, 5000 permutations) in the contralateral primary sensorimotor cortex (M1), supplementary motor area (SMA), Rolandic operculum, thalamus, and ipsilateral cerebellum. This activation pattern was seen on VPT and TC for both right- and left-hand tasks independently, except for the left-hand task on TC (n = 12), where activations were observed only in the contralateral M1 and ipsilateral cerebellum. Fig. 1 illustrates the within-group second-level analysis of each motor task, with detailed activation clusters in Table S2 of the Supplementary Material.

#### 3.2.3 Between-Group motor task fMRI comparison

##### 3.2.3.1 Comparison of BOLD activation pattern during hand tapping task

In the motor task fMRI comparison between preterm and term-born children, analysis of BOLD activation maps during hand tapping tasks revealed no significant differences between groups, regardless of whether the right (R) or left hand (L) was used. Specifically, the total activation volumes (cm^3^) for both hands were similar across the cohorts (p > 0.05). Activation strengths (t-value) in the Primary Motor Cortex (M1) were also comparable between the preterm and term-born groups for both hands (p > 0.05). Additionally, the volume of activation (cm^3^) within M1 did not display marked differences between the groups for either hand (R: p = 0.42; L: p = 0.99) during these hand-tapping motor tasks. For a breakdown of the BOLD activation patterns between preterm and term-born children, please refer to Table S3 in the Supplementary Material.

##### 3.2.3.2 Lateralisation of the BOLD response during motor tapping

In brain lobes that exhibited motor activation during hand tapping (Frontal, Parietal, Cerebellum), the LI followed canonical motor organisation, favouring the cortex contralateral to the moving hand and the ipsilateral cerebellar hemisphere. In contrast, lobes without significant activation still showed group-specific signal biases. To investigate these subtle effects, we used the LI method that is calculated from the full distribution of voxel-wise BOLD values within each lobe. This approach measures hemisphere-wide signal balance and is independent of activation thresholds, making it sensitive to systematic hemispheric asymmetries that might otherwise be missed.

**Fig. 2:**
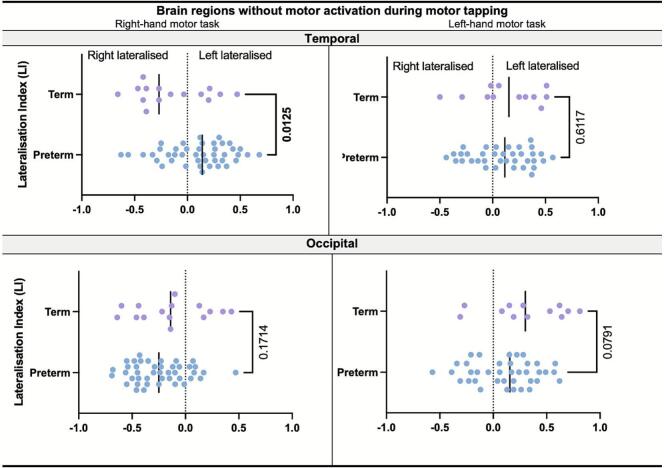
Scatter plot of the Lateralisation Indices (LI) for right and left-hand motor tapping (columns) on each respective brain lobe. Each point represents an individual subject, illustrating the distribution and trend of lateralisation within each group. The LI quantifies hemispheric signal balance, where positive values indicate left lateralisation and negative values indicate right lateralisation. LI was similar for brain lobes showing BOLD activation during tasks. On brain lobes without activation, preterm subjects show contralateral left lateralisation in the temporal lobe during right-hand motor task compared to term control.

During right-hand tapping, the VPT cohort displayed a significant left-ward lateralisation in the temporal lobe, reflected by predominantly positive LI values, whereas term controls showed a right-ward bias, reflected by predominantly negative LI values (p = 0.0125 Bonferroni-corrected; Fig. 2). Given the threshold-independent nature of the LI calculation, this temporal asymmetry reflects a global BOLD asymmetry rather than task-evoked activity in a specific, localised cluster. These findings were robust even when examining a sub-cohort of n = 34 participants with complete data across all fMRI modalities (see Table S2 and Figs. S2 and S3 of the supplementary information for detailed lateralisation indices). Both groups exhibited comparable lateralisation in the occipital lobe during both motor tasks.

##### 3.2.3.3 Whole brain activation differences between VPT and TC during hand tapping task (VPT > TC)

**Fig. 3:**
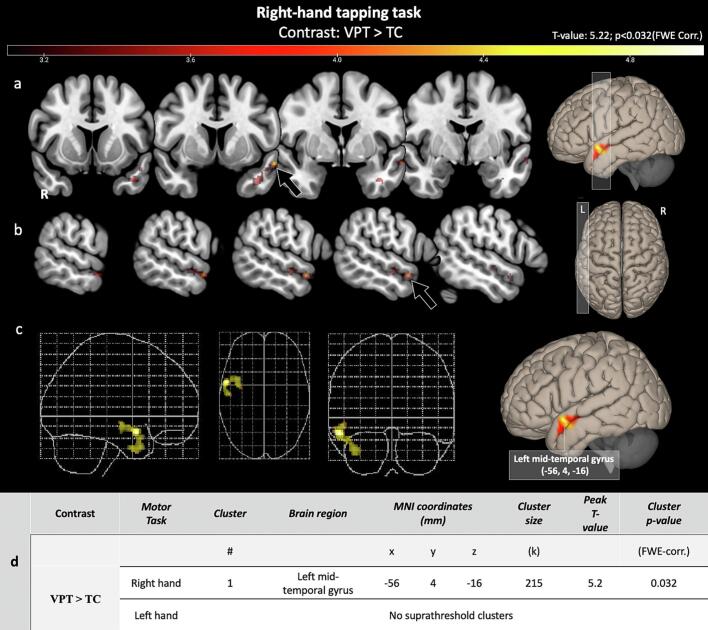
**Differential Motor Task Activation: VPT vs. TC.** This figure contrasts the very preterm (VPT) and term control (TC) groups during motor tasks. The VPT group exhibited heightened activation in the left superior and mid-left temporal gyrus during right-hand tapping (arrow) (MNI peak coordinates: −56, 4, −16). Adjustments were made for age at MRI, sex, handedness, and framewise displacement. Panels (a) and (b) display coronal and sagittal views. Panel (c) provides a glass brain and volume render, highlighting activation differences. Panel (d) details regions, cluster metrics, and significance. No suprathreshold clusters were found for left-hand tapping.

Compared to control participants, the VPT group displayed increased brain activation in the left superior and middle temporal gyrus region with MNI peak coordinates (−56, 4, −16) during right-hand motor tapping (Two-sample *t*-test with 5,000 permutations adjusted for age, sex, handedness, and FD). Fig. 3 illustrates task-based fMRI results for right- and left-hand motor tapping among VPT and TC. No differences in BOLD activation were found during the left motor tapping.

### 3.3 Resting-State fMRI results

We first compared ROI‑to‑ROI connectivity between PREBO‑6 term controls (n = 12) and external controls from ABIDE‑II + HBN (n = 40) using the study GLM (covariates: age, sex at birth, handedness, mean FD, site) with cluster-level inferences based on randomisation/permutation. No cortical/cerebellar or subcortical differences survived correction. With site retained as a covariate, we pooled controls to increase power and report two sets: PREBO‑6 (original cohort only) and PREBO‑6+ (original cohort plus external controls). Because ABIDE‑II and HBN do not exclude preterm birth or record gestational age, we assume very‑preterm (<32  weeks) prevalence approximates the population rate ∼1.5 % (Ohuma et al., 2023). All pooled results are labelled PREBO‑6+; external‑control criteria and sources are detailed in the Supplementary Material.

For both the PREBO-6 and PREBO-6+ analyses, the VPT and TC groups were well-matched regarding sex and handedness. However, in the PREBO-6+, the VPT group was approximately six months younger at the time of MRI acquisition compared to the combined term controls when including ABIDE-II and HBN datasets (6.1 years corrected age (IQR: 6–6.3 years) vs 6.6 years (IQR: 6.3–6.8 years); p < 0.0001). Despite this group difference, all children from both the PREBO-6 and external cohorts were scanned within the narrow six-year-old age range. Specific details on gestational age, birth weight, and other relevant metrics are presented in Supplementary Table S5. Motion metrics indicated that the framewise displacement (FD) was smaller for the VPT group compared to the TC group (FD: 0.2 vs. 0.3 mm; p = 0.024. These demographic differences, along with motion (FD) and data acquisition site, were included as covariates in subsequent GLM analyses to control for their potential influence.

#### 3.3.1 Subcortical ROI-to-ROI functional connectivity analysis

**Fig. 4:**
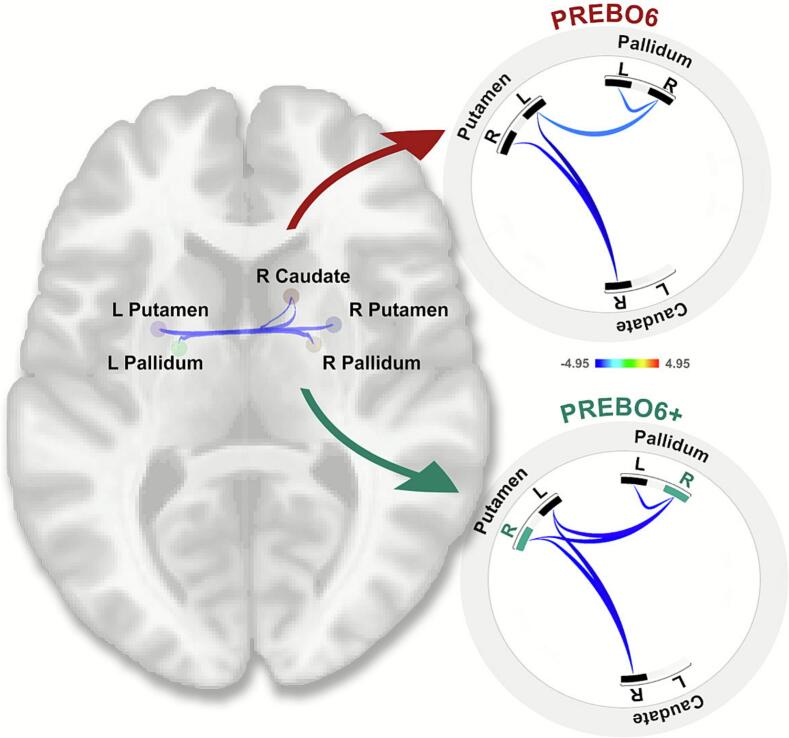
Altered Subcortical Connectivity Between Very Preterm and Term-born Groups. Group differences on contrast VPT > TC in resting-state functional connectivity of subcortical structures. The ROI-to-ROI results are presented using non-parametric permutation analyses with a combination of an uncorrected p < 0.01 height threshold, and an FDR-corrected p < 0.05 cluster-level threshold to select significant clusters (Zalesky et al., 2012). Left: Axial brain slice showing the spatial distribution and significantly weaker connectivity (blue lines) between subcortical ROIs (spheres). Top right: Connectome ring of PREBO-6 data depicting altered subcortical connectivity with weaker connectivity (blue lines) between caudate putamen and pallidum and decreased connectivity within the pallidum. Bottom right: Connectome right of PREBO-6+ analysis showing a similar connectivity pattern as PREBO-6 and additional weaker connectivity between right pallidum and right putamen was seen when including additional controls (PREBO-6+ ). (For interpretation of the references to colour in this figure legend, the reader is referred to the web version of this article.)

At the subcortical level, VPT participants had weaker connectivity between striatal structures, including the putamen, caudate, and pallidum, compared to TC participants (see Table 2). The inclusion of additional controls from the HBN and ABIDE-II datasets showed a similar pattern, with additional weaker connectivity observed between the right pallidum and right putamen (see Fig. 4).

**Table 2:** Subcortical ROI-to-ROI functional connectivity differences between children born very preterm (VPT) and term-born controls (TC). VPT participants show significantly weaker connectivity across basal ganglia nodes, including the putamen, caudate, and pallidum, and this pattern enlarges when the additional controls in the PREBO-6+ analysis are incorporated. Hemispheric labels: R, right; L, left. Abbreviations: p-unc. = uncorrected p-value; VPT-very preterm; TC-term control. For a graphical depiction, see Fig. 4. Subcortical Connectivity Between VPT and Term-born.

	**PREBO-6** VPT (n = 28) vs TC (n = 12)					**PREBO-6+** VPT (n = 28) vs TC (n = 52)				
**Functional connectivity weaker in VPT vs. term-born group**										
Clusters and connections	Connection	Statistic	p-unc.	p-FDR	Clusters and connections	Connection	Statistic	p-unc.	p-FDR	
**Cluster 1/2**		**Mass = 74.92**	**0.01**	**0.004**	**Cluster 1/2**		**Mass = 60.06**	**0.038**	**0.012**	
1	Caudate R − Putamen L	T(34) = -4.95	0.00	0.001	1	Pallidum R − Putamen L	T(73) = -3.55	0.001	0.015	
2	Caudate R − Putamen R	T(34) = -3.60	0.001	0.02	2	Pallidum R − Putamen R	T(73) = -3.15	0.002	0.036	
					3	Pallidum R − Pallidum L	T(73) = -2.75	0.008	0.057	
**Cluster 2/2**		**Mass = 34.74**	**0.0378**	**0.046**	**Cluster 2/2**		**Mass = 46.77**	**0.0383**	**0.023**	
3	Pallidum R − Pallidum L	T(34) = -2.98	0.005	0.07	4	Caudate R − Putamen L	T(73) = -3.89	0.000	0.010	
4	Pallidum R − Putamen L	T(34) = -2.92	0.006	0.07	5	Caudate R − Putamen R	T(73) = -2.87	0.005	0.057	
**Functional connectivity stronger in VPT vs term-born group**										
**Cluster 0/0**	**−**	**−**	**−**	**−**	**Cluster 0/0**	**−**	**−**	**−**	**−**

#### 3.3.2 Cortical and cerebellar ROI-to-ROI functional connectivity analysis

**Fig. 5:**
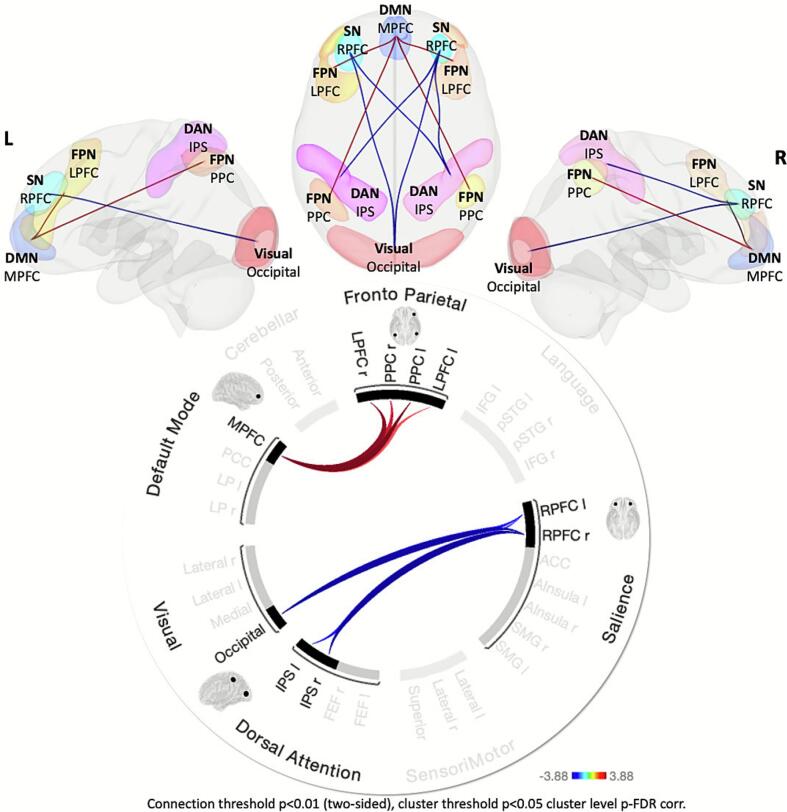
**Cortical resting-state Functional Connectivity Alterations in 6-Year-Old Very Preterm Children.** ROI-to-ROI analysis showing significant cortical functional connectivity group differences with contrast [VPT > TC]. The preterm group had weaker connectivity between the salience network (left and right RPFC) and the dorsal attention network (bilateral IPS), and between the salience network (bilateral RPFC) and the visual network (occipital region, blue lines), but stronger connectivity (decreased anticorrelation) between the MPFC and all elements of the frontal parietal network (red lines). Cerebellar, sensorimotor, and language networks at rest (in grey) showed no significant differences between both groups. p < 0.05, FDR corrected). (For interpretation of the references to colour in this figure legend, the reader is referred to the web version of this article.)

At the cortical and cerebellar level, VPT and TC groups revealed FC differences between resting-state networks, after controlling for age at scan, sex, handedness, and FD (Fig. 5). In the extended PREBO6 + analysis (n = 28 for preterm vs. n = 52 for term) (see Table 3), significant functional connectivity differences emerged. Specifically, the VPT group showed weaker inter-network connectivity compared to the term-born group between the salience network (left and right rostrolateral prefrontal cortex, RPFC) and the dorsal attention network (left and right intraparietal sulcus, IPS), and between the salience network (left and right RPFC) and the visual network (occipital) (p-FDR < 0.04). Conversely, the VPT group showed increased connectivity, reflecting decreased anticorrelation, between the frontal-parietal network (FPN; left and right lateral prefrontal cortex, LPFC, and posterior parietal cortex, PPC) and the medial prefrontal cortex (MPFC) of the default mode network (DMN) (p-FDR < 0.04), as indicated by red lines in Fig. 5.

**Table 3:** Resting-state functional connectivity contrasts between the PREBO-6 and PREBO-6 + analysis. Connection clusters marked with (*) show significant differences after FDR correction, while entries labelled “(ns)” did not reach significance after multiple comparison corrections.

	**PREBO-6**Group difference in connectivity strength preterm (n = 28) vs term (n = 12)					**PREBO-6+**Group difference in connectivity strength preterm (n = 28) vs term (n = 52)				
**Functional connectivity weaker in VPT vs. term-born group**										
Clusters and connections	Connection	Statistic	p-unc.	p-FDR	Clusters and connections	Connection	Statistic	p-unc.	p-FDR	
**1 Cluster**		**Mass = 98.58**	**0.077**	**0.051 (ns)**	**Cluster 1**		**Mass = 106.14**	**0.004**	0.04*	
	DAN.IPS (R) − SAL.RPFC (R)	−3.88	0.000	0.225	1	DAN.IPS (R) − SAL.RPFC (R)	T(73) = -3.61	0.0006	0.06	
	DAN.IPS (L) − SAL.RPFC (R)	−3.61	0.001	0.241	2	VN.occipital − SAL.RPFC (R)	T(73) = -3.57	0.0006	0.06	
	DAN.IPS (R) − SAL.RPFC (L)	−3.3	0.002	0.271	3	DAN.IPS (L) − SAL.RPFC (R)	T(73) = -3.13	0.003	0.15	
	DAN.IPS (L) − SAL.RPFC (L)	−3.2	0.003	0.271	4	VN.occipital − SAL.RPFC (L)	T(73) = -3.05	0.003	0.15	

**Functional connectivity stronger in VPT vs term-born group**										

Clusters and connections	Connection	Statistic	p-unc.	p-FDR	Clusters and connections	Connection	Statistic	p-unc.	p-FDR	
**2 Cluster**		**Mass = 56.16**	**0.176**	**0.177 (ns)**	**Cluster 2**		**Mass = 91.51**	**0.008**	0.04*	
	FPN.PPC (L) − DMN.MPFC	3.21	0.003	0.271	1	FPN.PPC (L) − DMN.MPFC	T(73) = 3.85	0.0003	0.06	
	FPN.LPFC (R) − DMN.MPFC	3.16	0.003	0.271	2	FPN.LPFC (R) − DMN.MPFC	T(73) = 3.64	0.0005	0.06	
	FPN.PPC (R) − DMN.MPFC	2.79	0.009	0.341	3	FPN.PPC (R) − DMN.MPFC	T(73) = 3.16	0.002	0.15	
					4	FPN.LPFC (L) − DMN.MPFC	T(73) = 2.78	0.007	0.2	

In the PREBO-6 analysis (n = 28 preterm vs. n = 12 term), a similar pattern emerged but did not reach statistical significance after multiple comparison correction (p-FDR = 0.051 for weaker connectivity; p-FDR = 0.17 for stronger connectivity). An exploratory analysis of the PREBO-6 data, using the default CONN uncorrected threshold (connection threshold p < 0.01, two-sided; cluster threshold p < 0.05, uncorrected), showed consistent trends, including decreased inter-network connectivity between the salience network (left and right RPFC) and the dorsal attention network (left and right IPS), and decreased anticorrelation between the DMN (MPFC) and FPN. A detailed list of connections highlighting functional connectivity differences in both the PREBO-6 and extended PREBO-6+ analyses is provided in Table 3.

#### 3.3.3 Correlation analysis of Resting-State functional connectivity with clinical demographics

**Fig. 6:**
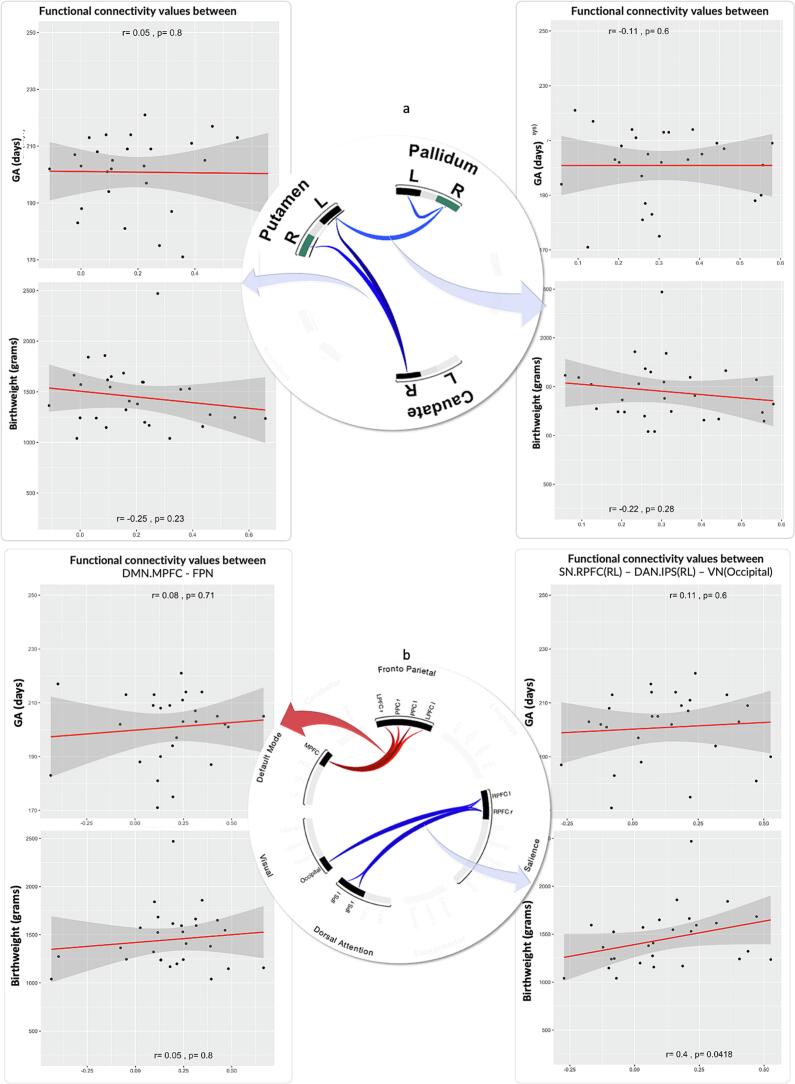
**Correlation between clinical demographics and functional connectivity measures.** Partial correlations between functional connectivity within cortical and subcortical networks and clinical measures of gestational age (GA) and birth weight, controlling for sex and age at scan. (a) Subcortical network: (b) Cortical resting-state networks.

At the subcortical level, we examined relationships between functional connectivity and clinical variables within the VPT group (Fig. 6a). Connectivity within subcortical circuits (right caudate-putamen and pallidum-putamen) did not show a significant relationship with either gestational age at birth or birth weight (all p > 0.22; see Supplementary Table S5 for full statistics).

At the cortical level (Fig. 6b), the atypical connectivity between the DMN and FPN did not correlate with either gestational age or birth weight. Conversely, the weakened connectivity between the salience, dorsal attention, and visual networks was related to birthweight. (r = 0.402, p = 0.04), where lower birth weight was associated with weaker connectivity. While this suggests a potential dose–response relationship, the finding did not remain significant after correcting for multiple comparisons.

## 4 Discussion

This study employed a dual-approach design, using both task-based and resting-state fMRI to investigate functional brain network differences between VPT and TC children at six years of age. Overall, we observed (1) increased activation and altered lateralisation in the left temporal lobe during a right-hand motor task in VPT children, despite similar primary motor cortex activation to TCs; (2) broadly weaker resting-state FC within subcortical networks (striatum) in the VPT group; (3) reduced resting-state FC between key cortical networks (Salience, Dorsal Attention, Visual) in VPT children; and (4) decreased anticorrelation between the DMN and FPN at rest in the VPT group. These findings point towards lasting neurodevelopmental reorganisation following VPT birth.

### 4.1 Atypical motor task activation following very preterm birth

Previous studies of finger tapping have demonstrated that comparable motor network regions are activated in typically developing children, adults, and individuals born VPT (F. De Guio et al., 2012, Lawrence et al., 2014, Turesky et al., 2018, Wuthrich et al., 2023). Our findings are consistent with these findings, showing similar activation in the primary sensorimotor cortex for both the VPT and term-born control groups. In our cohort, we likewise observed comparable activation in the contralateral primary sensorimotor cortex. However, group differences also emerged outside core motor areas. The increased recruitment of the left middle temporal gyrus specifically during right-hand tapping in VPT children suggests altered neural strategies. Such increased temporal lobe activation during motor tasks is characteristic of younger, less-developed motor systems (François De Guio et al., 2012). Notably, this pattern seems to continue into adulthood for those born VPT, as previous studies have shown increased activation of the middle temporal gyri in VPT adults during motor tasks compared to controls (Lawrence et al., 2014). The persistence of this activation profile into adulthood suggests a stable, long-term adaptation rather than merely a maturational delay in functional organisation.

The observed left-lateralised temporal activation during the right-hand task in our VPT cohort further reinforces the notion of altered neural strategies. Typical sensorimotor maturation involves a dynamic process: functional responses initially become more spatially dispersed through the late preterm period, engaging bilateral cortical areas and the SMA, before refining towards more focal, contralateral patterns around term (Allievi et al., 2016). Early extrauterine exposure likely disrupts this complex sequence of network integration and subsequent specialisation (Bouyssi-Kobar et al., 2019). The persistent recruitment of secondary regions, such as the temporal lobe, in our cohort at age six years may reflect an enduring consequence of this disrupted developmental trajectory. This could represent a compensatory mechanism, potentially driven by the dominant hemisphere specialised for motor planning (Sainburg, 2002). Such functional compensation could be necessitated by underlying structural differences, including reduced temporal lobe volume (Nosarti et al., 2008) and thickness (Lawrence et al., 2014). Furthermore, the interplay between structure and function is highlighted by findings that reduced premotor grey matter volume relates to increased functional secondary activation during motor tasks in VPT adults (Lawrence et al., 2014), suggesting that VPT individuals engage broader neuronal circuits to maintain performance within structural constraints.

### 4.2 Altered resting-state network connectivity

#### 4.2.1 Subcortical networks hypoconnectivity

The subcortical network, encompassing the striatum (putamen, caudate, pallidum) and thalamus, forms a critical circuit for motor and cognitive functions (Alexander et al., 1986, Lanciego et al., 2012, Robinson et al., 2009). While sometimes underrepresented in resting-state analyses, its intrinsic FC is reliably detectable from infancy through adulthood (Damaraju et al., 2010, Griffanti et al., 2018, Robinson et al., 2009, Szewczyk-Krolikowski et al., 2014). We focused on this network given prior structural and functional evidence indicating its vulnerability following preterm birth (Abernethy et al., 2004, Loh et al., 2017, Meng et al., 2016, Nosarti et al., 2014, Peterson et al., 2000, Srinivasan et al., 2007, Thompson et al., 2020, White et al.,2014).

Although the fundamental topography of major resting-state networks seems established by 18 months in both preterm and term infants (Damaraju et al., 2010), divergence in network properties emerges later in development. Specifically, by 36 months, preterm children exhibit altered basal ganglia spectral power characteristics and weaker inter-network functional connectivity compared to term controls (Damaraju et al., 2010). Our findings at age six align with this developmental alteration, revealing persistent subcortical functional changes, with weaker intrinsic FC within the striatum in VPT children relative to TCs.

This functional hypoconnectivity (i.e. reduced functional connectivity) in the striatum is paralleled by a well-documented trajectory of structural deficits. Meta-analyses and cohort studies have established that individuals born VPT exhibit persistent volume reductions in basal ganglia regions (Kelly et al., 2023). This pattern is observable across the lifespan, from infancy and early childhood (Abernethy et al., 2004, Loh et al., 2017, Peterson et al., 2000, Srinivasan et al., 2007) to adolescence (Nosarti et al., 2008) and adulthood (Lawrence et al., 2014, Meng et al., 2016, Nosarti et al., 2014). Functionally, this reduced striatal network connectivity extends into adulthood and is associated with executive function deficits in VPT individuals (T. P. White et al., 2014). The presence of such subcortical network hypoconnectivity in childhood following VPT birth stands in contrast to the typical lifespan pattern, where connectivity within these circuits generally remains robust until declining in later healthy aging (e.g., after age 60) (Griffanti et al., 2018). Interestingly, within our VPT sample, the degree of subcortical connectivity reduction was not significantly correlated with gestational age or birthweight, perhaps suggesting that early extrauterine environment exposure imposes a substantial functional change (Bouyssi-Kobar et al., 2019) rather than a graded effect based on clinical factors within this high-risk group.

#### 4.2.2 Atypical cortical network Interaction

Significant alterations were also identified in the functional interactions between large-scale cortical networks in VPT children. Normative development involves the progressive functional segregation of networks, particularly the strengthening of anticorrelation between the internally-oriented DMN and externally-oriented task-positive networks (SN, DAN, VN, FPN) (Chen et al., 2023, Whitfield-Gabrieli et al., 2020). This dynamic interplay is thought to support efficient cognitive control and behaviour (Kelly et al., 2008). Our VPT cohort displayed two primary deviations from this typical developmental course:

Weakened Task-Positive Network Coupling:

FC was significantly reduced between nodes of the SN (rostrolateral prefrontal cortex), DAN (intraparietal sulcus), and VN (occipital regions). These networks typically co-activate during tasks requiring external attention and sensory processing. The observed weakened coupling suggests less efficient integration across these key systems in VPT children, consistent with prior reports of altered or simplified connectivity patterns involving these networks in preterm cohorts (Cho et al., 2022, Smyser et al., 2016, Wehrle et al., 2018, Wheelock et al., 2018). The weakened coupling in VPT children suggests impaired integration of attentional and sensory systems, echoing prior findings in preterm populations (Cho et al., 2022, Smyser et al., 2016, Wehrle et al., 2018). Notably, this disruption emerges early: preterm infants display a simpler, less integrated network architecture (Smyser et al., 2016), and it persists into the school-age years. By early childhood, VPT individuals show reduced connectivity among the SMN, DAN, and visual networks (Wehrle et al., 2018, Wheelock et al., 2018).

Reduced Anticorrelation Between DMN and FPN:

The characteristic anticorrelation between the DMN (medial prefrontal cortex) and the FPN (lateral prefrontal/posterior parietal cortices) was significantly attenuated in VPT children. This reduced functional segregation deviates markedly from the typical trajectory (Chen et al., 2023, Whitfield-Gabrieli et al., 2020) and aligns consistently with findings in other preterm samples linking this pattern to poorer executive attention (Degnan et al., 2015, Wheelock et al., 2021). This may reflect an immature network configuration; DMN and FPN show positive functional coupling in utero which typically transitions towards anticorrelation postnatally (Scheinost et al., 2024). The persistence of a more integrated state in VPT children could hinder efficient switching between internal and external cognitive modes, contributing to attentional difficulties. The neurobiological substrate likely involves disruptions to maturational processes like synaptic pruning and myelination, potentially related to the increased risk of white matter injury in preterm infants, which are necessary for refining distinct network boundaries (Smyser et al., 2016).

### 4.3 Neurodevelopmental implications and plasticity

Collectively, these distinct patterns of altered functional connectivity, including weakened subcortical integration, impaired task-positive network coupling, and reduced DMN-FPN segregation, provide a neurobiological framework for understanding the common profile of attentional, motor, and cognitive challenges observed in children born VPT. Although individual prediction remains challenging (Sadraee et al., 2021), these findings emphasise the significant, enduring impact of preterm birth on the brain's functional organisation. Importantly, neural systems exhibit considerable plasticity. Evidence indicates that early interventions, such as optimising neonatal nutrition (Duerden et al., 2021), and later cognitive training approaches like mindfulness (Siffredi et al., 2023), can positively influence brain connectivity and functional outcomes in preterm populations. This underscores the potential for targeted interventions across development, potentially guided by neuroimaging insights, to mitigate adverse long-term consequences.

### 4.4 Strengths, limitations and future directions

We studied a homogeneous cohort of very preterm children assessed at a single time point (6 years) using a single-site protocol, along with term controls. Task and resting-state fMRI were acquired in the same session, and were analysed using non-parametric permutation tests with appropriate multiple-comparison control. Data quality was supported by appropriate motion QC and phase-reversed SE-EPI distortion correction.

Several limitations, however, warrant consideration. The initial resting-state analysis was constrained by a small and unbalanced term-control group (n = 12), potentially limiting statistical power and generalizability. While supplementing the analysis with external controls (n = 40) and covarying for site improved power and yielded consistent results, this approach does not fully replace a larger, prospectively recruited and balanced cohort. Methodologically, our use of template-based ROIs for resting-state network analysis aligns with recent work in similarly aged VPT children (Cho et al., 2022, Wehrle et al., 2018) but may not optimally capture individual functional variability inherent to this population; future work might explore data-driven ROI approaches, while carefully considering potential trade-offs in reproducibility. Furthermore, despite rigorous motion correction procedures including subject exclusion and covariate regression (Power et al., 2012), motion artifacts remain a significant challenge in paediatric fMRI, contributing to data loss, particularly during resting-state scans (see Supplementary Fig. S1). Exploring less demanding paradigms, such as naturalistic viewing, could improve compliance and data retention in future studies.

## 5 Conclusion

Utilising task-based and resting-state fMRI, this study identified significant alterations in functional brain activity and connectivity in six-year-old children born VPT. Compared to term-born controls, VPT children demonstrated atypical, potentially compensatory, recruitment of temporal regions during a motor task. Their intrinsic functional architecture at rest was characterised by hypoconnectivity within subcortical circuits and between key task-positive cortical networks (Salience, Dorsal Attention, Visual), coupled with reduced functional segregation between the DMN and FPN. These findings highlight the persistent influence of VPT birth on the developing functional connectome, offering potential neural substrates for the associated neurodevelopmental challenges.

## CRediT authorship contribution statement

**Javier Urriola:** Writing – review & editing, Writing – original draft, Visualization, Methodology, Investigation, Formal analysis, Conceptualization. **Kerstin Pannek:** Writing – review & editing, Methodology, Investigation. **DanaKai Bradford:** Writing – review & editing, Investigation. **Jurgen Fripp:** Writing – review & editing, Supervision, Methodology, Investigation. **Julie Trinder:** Investigation. **Karen Mistry:** Investigation. **Paul B Colditz:** Writing – review & editing, Conceptualization. **Roslyn Boyd:** Writing – review & editing, Supervision, Conceptualization. **Samudragupta Bora:** Writing – review & editing, Funding acquisition, Conceptualization. **Joanne George:** Writing – review & editing, Project administration, Investigation, Funding acquisition, Conceptualization. **Alex Pagnozzi:** Writing – review & editing, Investigation, Funding acquisition, Formal analysis, Conceptualization.

## Declaration of Competing Interest

The authors declare that they have no known competing financial interests or personal relationships that could have appeared to influence the work reported in this paper.

## Data Availability

The authors do not have permission to share data.

## References

[b0005] Abernethy L.J., Cooke R.W., Foulder-Hughes L. (2004). Caudate and hippocampal volumes, intelligence, and motor impairment in 7-year-old children who were born preterm. Pediatr. Res..

[b0010] Alexander G.E., DeLong M.R., Strick P.L. (1986). Parallel organization of functionally segregated circuits linking basal ganglia and cortex. Annu. Rev. Neurosci..

[b0015] Alexander L.M., Escalera J., Ai L., Andreotti C., Febre K., Mangone A., Vega-Potler N., Langer N., Alexander A., Kovacs M., Litke S., O'Hagan B., Andersen J., Bronstein B., Bui A., Bushey M., Butler H., Castagna V., Camacho N., Milham M.P. (2017). An open resource for transdiagnostic research in pediatric mental health and learning disorders. Sci. Data.

[b0020] Allievi A.G., Arichi T., Tusor N., Kimpton J., Arulkumaran S., Counsell S.J., Edwards A.D., Burdet E. (2016). Maturation of sensori-motor functional responses in the preterm brain. Cereb. Cortex.

[b0025] Allin M.P., Kontis D., Walshe M., Wyatt J., Barker G.J., Kanaan R.A., McGuire P., Rifkin L., Murray R.M., Nosarti C. (2011). White matter and cognition in adults who were born preterm. PLoS One.

[b0030] Anderson, P. J., de Miranda, D. M., Albuquerque, M. R., Indredavik, M. S., Evensen, K. A. I., Van Lieshout, R., Saigal, S., Taylor, H. G., Raikkonen, K., Kajantie, E., Marlow, N., Johnson, S., Woodward, L. J., Austin, N., Nosarti, C., Jaekel, J., Wolke, D., Cheong, J. L. Y., Burnett, A.,…Doyle, L. W., 2021. Psychiatric disorders in individuals born very preterm / very low-birth weight: An individual participant data (IPD) meta-analysis. eClinicalMedicine, 42. doi: 10.1016/j.eclinm.2021.101216. 10.1016/j.eclinm.2021.101216 PMC8639417 34901794

[b0035] Behzadi Y., Restom K., Liau J., Liu T.T. (2007). A component based noise correction method (CompCor) for BOLD and perfusion based fMRI. Neuroimage.

[b0040] Bouyssi-Kobar M., De Asis-Cruz J., Murnick J., Chang T., Limperopoulos C. (2019). Altered functional brain network integration, segregation, and modularity in infants born very preterm at term-equivalent age. J. Pediatr..

[b0045] Chen M., He Y., Hao L., Xu J., Tian T., Peng S., Zhao G., Lu J., Zhao Y., Zhao H. (2023). Default mode network scaffolds immature frontoparietal network in cognitive development. Cereb. Cortex.

[b0050] Cheong J.L.Y., Anderson P.J., Burnett A.C., Roberts G., Davis N., Hickey L., Carse E., Doyle L.W., Group, f. t. V. I. C. S (2017). Changing neurodevelopment at 8 years in children born extremely preterm since the 1990s. Pediatrics.

[b0055] Cheong J.L.Y., Mainzer R.M., Doyle L.W., Olsen J.E., Ellis R., FitzGerald T.L., Cameron K.L., Rossetti L., Anderson P.J., Spittle A.J. (2024). Neurodevelopment at age 9 years among children born at 32 to 36 weeks' gestation. JAMA Netw. Open.

[b0060] Cho H.J., Jeong H., Park C.A., Son D.W., Shim S.Y. (2022). Altered functional connectivity in children born very preterm at school age. Sci. Rep..

[b0065] Damaraju E., Phillips J.R., Lowe J.R., Ohls R., Calhoun V.D., Caprihan A. (2010). Resting-state functional connectivity differences in premature children. Front. Syst. Neurosci..

[b0070] De Guio F., Jacobson S.W., Molteno C.D., Jacobson J.L., Meintjes E.M. (2012). Functional magnetic resonance imaging study comparing rhythmic finger tapping in children and adults. Pediatr. Neurol..

[b0075] Degnan A.J., Wisnowski J.L., Choi S., Ceschin R., Bhushan C., Leahy R.M., Corby P., Schmithorst V.J., Panigrahy A. (2015). Altered structural and functional connectivity in late preterm preadolescence: an anatomic seed-based study of resting state networks related to the posteromedial and lateral parietal cortex. PLoS One.

[b0080] Di Martino A., O'Connor D., Chen B., Alaerts K., Anderson J.S., Assaf M., Balsters J.H., Baxter L., Beggiato A., Bernaerts S., Blanken L.M., Bookheimer S.Y., Braden B.B., Byrge L., Castellanos F.X., Dapretto M., Delorme R., Fair D.A., Fishman I., Milham M.P. (2017). Enhancing studies of the connectome in autism using the autism brain imaging data exchange II. Sci. Data.

[b0085] Duerden E.G., Thompson B., Poppe T., Alsweiler J., Gamble G., Jiang Y., Leung M., Tottman A.C., Wouldes T., Miller S.P., Harding J.E., group, P.s. (2021). Early protein intake predicts functional connectivity and neurocognition in preterm born children. Sci. Rep..

[b0090] Edwards J., Berube M., Erlandson K., Haug S., Johnstone H., Meagher M., Sarkodee-Adoo S., Zwicker J.G. (2011). Developmental coordination disorder in school-aged children born very preterm and/or at very low birth weight: a systematic review. J. Dev. Behav. Pediatr..

[b0095] Eikenes L., Løhaugen G.C., Brubakk A.-M., Skranes J., Håberg A.K. (2011). Young adults born preterm with very low birth weight demonstrate widespread white matter alterations on brain DTI. Neuroimage.

[b0100] Fawke, J. (2007). Neurological outcomes following preterm birth. Seminars in fetal and neonatal medicine. 10.1016/j.siny.2007.06.002 17719861

[b0105] Fox M.D., Raichle M.E. (2007). Spontaneous fluctuations in brain activity observed with functional magnetic resonance imaging. Nat. Rev. Neurosci..

[b0110] George J.M., Boyd R.N., Colditz P.B., Rose S.E., Pannek K., Fripp J., Lingwood B.E., Lai M.M., Kong A.H., Ware R.S., Coulthard A., Finn C.M., Bandaranayake S.E. (2015). PPREMO: a prospective cohort study of preterm infant brain structure and function to predict neurodevelopmental outcome. BMC Pediatr..

[b0115] George J.M., Pagnozzi A.M., Bora S., Boyd R.N., Colditz P.B., Rose S.E., Ware R.S., Pannek K., Bursle J.E., Fripp J., Barlow K., Iyer K., Leishman S.J., Jendra R.L. (2020). Prediction of childhood brain outcomes in infants born preterm using neonatal MRI and concurrent clinical biomarkers (PREBO-6): study protocol for a prospective cohort study. BMJ Open.

[b0120] Griffanti L., Stratmann P., Rolinski M., Filippini N., Zsoldos E., Mahmood A., Zamboni G., Douaud G., Klein J.C., Kivimäki M. (2018). Exploring variability in basal ganglia connectivity with functional MRI in healthy aging. Brain Imaging Behav..

[b0125] Hee Chung E., Chou J., Brown K.A. (2020). Neurodevelopmental outcomes of preterm infants: a recent literature review. Transl. Pediatr..

[b0130] Kassambara, A., 2018. ggpubr:'ggplot2'based publication ready plots. *R package version*, 2.

[b0135] Kelly A.C., Uddin L.Q., Biswal B.B., Castellanos F.X., Milham M.P. (2008). Competition between functional brain networks mediates behavioral variability. Neuroimage.

[b0140] Kelly C.E., Shaul M., Thompson D.K., Mainzer R.M., Yang J.Y., Dhollander T., Cheong J.L., Inder T.E., Doyle L.W., Anderson P.J. (2023). Long-lasting effects of very preterm birth on brain structure in adulthood: a systematic review and meta-analysis. Neurosci. Biobehav. Rev..

[b0145] Lanciego J.L., Luquin N., Obeso J.A. (2012). Functional neuroanatomy of the basal ganglia. Cold Spring Harb. Perspect. Med..

[b0150] Lawrence E.J., Froudist-Walsh S., Neilan R., Nam K.W., Giampietro V., McGuire P., Murray R.M., Nosarti C. (2014). Motor fMRI and cortical grey matter volume in adults born very preterm. Dev. Cogn. Neurosci..

[b0155] Loh W.Y., Anderson P.J., Cheong J.L.Y., Spittle A.J., Chen J., Lee K.J., Molesworth C., Inder T.E., Connelly A., Doyle L.W., Thompson D.K. (2017). Neonatal basal ganglia and thalamic volumes: very preterm birth and 7-year neurodevelopmental outcomes. Pediatr. Res..

[b0160] McHaffie J.G., Stanford T.R., Stein B.E., Coizet V., Redgrave P. (2005). Subcortical loops through the basal ganglia. Trends Neurosci..

[b0165] Meng C., Bäuml J.G., Daamen M., Jaekel J., Neitzel J., Scheef L., Busch B., Baumann N., Boecker H., Zimmer C. (2016). Extensive and interrelated subcortical white and gray matter alterations in preterm-born adults. Brain Struct. Funct..

[b0170] Nosarti C., Giouroukou E., Healy E., Rifkin L., Walshe M., Reichenberg A., Chitnis X., Williams S.C., Murray R.M. (2008). Grey and white matter distribution in very preterm adolescents mediates neurodevelopmental outcome. Brain.

[b0175] Nosarti C., Nam K.W., Walshe M., Murray R.M., Cuddy M., Rifkin L., Allin M.P. (2014). Preterm birth and structural brain alterations in early adulthood. Neuroimage Clin..

[b0180] Ohuma E.O., Moller A.B., Bradley E., Chakwera S., Hussain-Alkhateeb L., Lewin A., Okwaraji Y.B., Mahanani W.R., Johansson E.W., Lavin T., Fernandez D.E., Dominguez G.G., de Costa A., Cresswell J.A., Krasevec J., Lawn J.E., Blencowe H., Requejo J., Moran A.C. (2023). National, regional, and global estimates of preterm birth in 2020, with trends from 2010: a systematic analysis. Lancet.

[b0185] Peterson B.S., Vohr B., Staib L.H., Cannistraci C.J., Dolberg A., Schneider K.C., Katz K.H., Westerveld M., Sparrow S., Anderson A.W., Duncan C.C., Makuch R.W., Gore J.C., Ment L.R. (2000). Regional brain volume abnormalities and long-term cognitive outcome in preterm infants. J. Am. Med. Assoc..

[b0190] Poldrack R.A., Mumford J.A., Nichols T.E. (2011).

[b0195] Power J.D., Barnes K.A., Snyder A.Z., Schlaggar B.L., Petersen S.E. (2012). Spurious but systematic correlations in functional connectivity MRI networks arise from subject motion. Neuroimage.

[b0200] R Core Team. (2021). *A language and environment for statistical computing*. In R Foundation for Statistical Computing, Vienna, Austria. https://www.R-project.org/.

[b0205] Robinson S., Basso G., Soldati N., Sailer U., Jovicich J., Bruzzone L., Kryspin-Exner I., Bauer H., Moser E. (2009). A resting state network in the motor control circuit of the basal ganglia. BMC Neurosci..

[b0210] Rogers C.E., Lean R.E., Wheelock M.D., Smyser C.D. (2018). Aberrant structural and functional connectivity and neurodevelopmental impairment in preterm children. J. Neurodev. Disord..

[b0215] Sadraee A., Paulus M., Ekhtiari H. (2021). fMRI as an outcome measure in clinical trials: a systematic review in clinicaltrials.gov. Brain Behav..

[b0220] Sainburg R.L. (2002). Evidence for a dynamic-dominance hypothesis of handedness. Exp. Brain Res..

[b0225] Satterthwaite T.D., Elliott M.A., Gerraty R.T., Ruparel K., Loughead J., Calkins M.E., Eickhoff S.B., Hakonarson H., Gur R.C., Gur R.E., Wolf D.H. (2013). An improved framework for confound regression and filtering for control of motion artifact in the preprocessing of resting-state functional connectivity data. Neuroimage.

[b0230] Scheinost D., Chang J., Brennan-Wydra E., Lacadie C., Constable R.T., Chawarska K., Ment L.R. (2024). Developmental trajectories of the default mode, frontoparietal, and salience networks from the third trimester through the newborn period. Imaging Neurosci..

[b0235] Siegel J.S., Power J.D., Dubis J.W., Vogel A.C., Church J.A., Schlaggar B.L., Petersen S.E. (2014). Statistical improvements in functional magnetic resonance imaging analyses produced by censoring high-motion data points. Hum. Brain Mapp..

[b0240] Siffredi V., Liverani M.C., Freitas L.G., Tadros D., Farouj Y., Borradori Tolsa C., Van De Ville D., Hüppi P.S., Ha-Vinh Leuchter R. (2023). Large-scale brain network dynamics in very preterm children and relationship with socio-emotional outcomes: an exploratory study. Pediatr. Res..

[b0245] Smyser C.D., Snyder A.Z., Shimony J.S., Mitra A., Inder T.E., Neil J.J. (2016). Resting-State network complexity and magnitude are reduced in prematurely born infants. Cereb. Cortex.

[b0250] Spittle A.J., Orton J. (2014). Cerebral palsy and developmental coordination disorder in children born preterm. Semin. Fetal Neonatal Med..

[b0255] Srinivasan L., Dutta R., Counsell S.J., Allsop J.M., Boardman J.P., Rutherford M.A., Edwards A.D. (2007). Quantification of deep gray matter in preterm infants at term-equivalent age using manual volumetry of 3-tesla magnetic resonance images. Pediatrics.

[b0260] Szewczyk-Krolikowski K., Menke R.A., Rolinski M., Duff E., Salimi-Khorshidi G., Filippini N., Zamboni G., Hu M.T., Mackay C.E. (2014). Functional connectivity in the basal ganglia network differentiates PD patients from controls. Neurology.

[b0265] Thompson D.K., Loh W.Y., Connelly A., Cheong J.L., Spittle A.J., Chen J., Kelly C.E., Inder T.E., Doyle L.W., Anderson P.J. (2020). Basal ganglia and thalamic tract connectivity in very preterm and full-term children; associations with 7-year neurodevelopment. Pediatr. Res..

[b0270] Tokariev M., Vuontela V., Tokariev A., Lonnberg P., Andersson S., Maenpaa H., Metsaranta M., Lano A., Carlson S. (2025). Imprints of extreme prematurity on functional brain networks in school-aged children and adolescents. Neuroimage.

[b0275] Turesky T.K., Olulade O.A., Luetje M.M., Eden G.F. (2018). An fMRI study of finger tapping in children and adults. Hum. Brain Mapp..

[b0280] Uusitalo K., Haataja L., Saunavaara V., Lind A., Vorobyev V., Tilli J., Parkkola R., Group, P.S (2021). Performance in hand coordination tasks and concurrent functional MRI findings in 13-year-olds born very preterm. Pediatr. Neurol..

[b0285] Wehrle F.M., Michels L., Guggenberger R., Huber R., Latal B., O'Gorman R.L., Hagmann C.F. (2018). Altered resting-state functional connectivity in children and adolescents born very preterm short title. Neuroimage Clin..

[b0290] Weyandt L.L., Clarkin C.M., Holding E.Z., May S.E., Marraccini M.E., Gudmundsdottir B.G., Shepard E., Thompson L. (2020). Neuroplasticity in children and adolescents in response to treatment intervention: a systematic review of the literature. Clin. Trans. Neurosci..

[b0295] Wheelock M.D., Austin N.C., Bora S., Eggebrecht A.T., Melzer T.R., Woodward L.J., Smyser C.D. (2018). Altered functional network connectivity relates to motor development in children born very preterm. Neuroimage.

[b0300] Wheelock M.D., Lean R.E., Bora S., Melzer T.R., Eggebrecht A.T., Smyser C.D., Woodward L.J. (2021). Functional connectivity network disruption underlies domain-specific impairments in attention for children born very preterm. Cereb. Cortex.

[b0320] Whitfield-Gabrieli S., Nieto-Castanon A. (2012). Conn: a functional connectivity toolbox for correlated and anticorrelated brain networks. Brain Connect..

[b0325] Whitfield-Gabrieli S., Wendelken C., Nieto-Castañón A., Bailey S.K., Anteraper S.A., Lee Y.J., Chai X.-Q., Hirshfeld-Becker D.R., Biederman J., Cutting L.E. (2020). Association of intrinsic brain architecture with changes in attentional and mood symptoms during development. JAMA Psychiat..

[b9020] White T., Muetzel R., Schmidt M., Langeslag S.J., Jaddoe V., Hofman A., Calhoun V.D., Verhulst V.D., Tiemeier H. (2014). Time of acquisition and network stability in pediatric resting-state functional magnetic resonance imaging. Brain Connectivity.

[b9025] White T.P. (2014). Dysconnectivity of neurocognitive networks at rest in very-preterm born adults. NeuroImage: Clinical.

[b0330] Wilke M., Schmithorst V.J. (2006). A combined bootstrap/histogram analysis approach for computing a lateralization index from neuroimaging data. Neuroimage.

[b0335] Wuthrich F., Lefebvre S., Nadesalingam N., Bernard J.A., Mittal V.A., Shankman S.A., Walther S. (2023). Test-retest reliability of a finger-tapping fMRI task in a healthy population. Eur. J. Neurosci..

[b0340] Zalesky A., Fornito A., Bullmore E. (2012). On the use of correlation as a measure of network connectivity. Neuroimage.

[b0345] Zhang Y., Inder T.E., Neil J.J., Dierker D.L., Alexopoulos D., Anderson P.J., Van Essen D.C. (2015). Cortical structural abnormalities in very preterm children at 7 years of age. Neuroimage.

