# Supplementary material for: Altered functional brain organisation in preterm Children: Motor task and resting-state fMRI findings at six years
Source: Neuroimage Clin. 2025 Nov 8;48:103906. doi: 10.1016/j.nicl.2025.103906 (PMC12671035; doi:10.1016/j.nicl.2025.103906)
Supplement: Supplementary Data 1 [file mmc1.docx]

# Supplementary Material

## Flowchart of Participant Recruitment and Data Inclusion for fMRI Analyses.

| 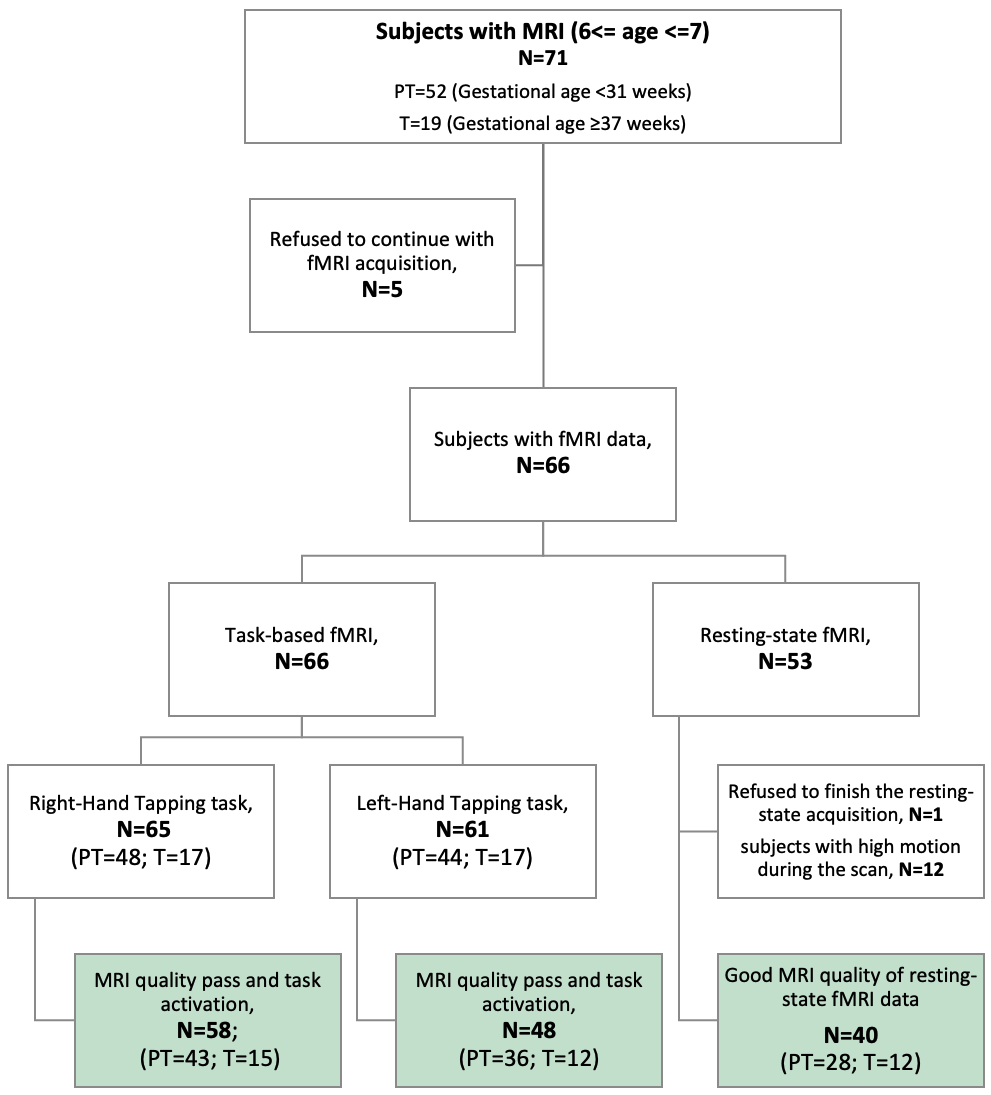 |
| --- |
| **Supplementary figure S1**: The flowchart outlines the participant selection process and data availability in the study acquisitions. Initially, 71 participants were enrolled, with 66 providing usable fMRI data. Task-based fMRI was performed by all 66 participants, with 65 completing a right-hand tapping task and 61 completing a left-hand tapping task. Quality control measures resulted in 58 participants passing for the right-hand task and 48 for the left-hand task. Resting-state fMRI data was available for 53 participants, with 40 passing quality control for motion. The flowchart also indicates the number of participants who refused to continue with certain parts of the study and those excluded due to high motion during scans. (Flowchart will be added in supplementary information). |

## Additional control groups for rs-fMRI analysis

To increase the statistical power of our resting-state analysis, additional healthy controls were obtained from two large, publicly available imaging databases: the Autism Brain Imaging Data Exchange (ABIDE-II) (Di Martino et al., 2017) and the Healthy Brain Network (HBN) Biobank (Alexander et al., 2017; releases 3-10).

Inclusion criteria for the external control cohort were: (1) age at MRI matching our PREBO-6 cohort (5.9 > 7.0 years inclusive); (2) availability of at least one T1-weighted anatomical scan; and (3) a 3T resting-state T2* Gradient Echo (GRE) Echo Planar Imaging (EPI) acquisition of at least 6 minutes, with a minimum of 4 minutes of usable data remaining after quality control and motion censoring. Healthy controls were defined as individuals without a history of neurological or psychiatric disorders, as determined by semi-structured interviews and parent/self-reports.

As the MRI acquisition parameters were not identical across sites (see Table S1), potential biases were controlled by including 'data acquisition site' as a nuisance covariate in all group-level statistical models, as detailed in the main manuscript (Methods section 2.3.2). This approach was used to ensure that the findings reflect biological group differences rather than scanner-specific artifacts.

## MRI Scan Parameters

Key acquisition parameters for the resting-state fMRI scans from each external cohort are detailed in Table S1 below.

| Dataset | Collection Label | MRI | TR (ms) | TE (ms) | Flip Angle (°) | Voxel Size (mm) | Matrix | Scan Duration (min:sec) |
| --- | --- | --- | --- | --- | --- | --- | --- | --- |
| ABIDE-II | EMC_1 | 3T GE MR750 | 2000 | 30 | 85 | 3.59 x 3.59 x 4 | 64x64x37 | 5:20 |
| ABIDE-II | NYU_1 | 3T Siemens Allegra | 2000 | 15 | 90 | 3 x 3 x 4 | 80x64x33 | 6:00 |
| HBN | CitiGroup Cornell | 3T Siemens Prisma | 800 | 30 | 31 | 2.4 x 2.4 x 2.4 | 92x92x60 | 6:20 |

**Table S1** - Full parameter definitions are available in the respective original publications (Alexander et al., 2017; Di Martino et al., 2017)

*ABIDE-II dataset*: Seven term-born controls were drawn from the EMC_1 (Erasmus University Medical Center, Rotterdam) and NYU_1 (NYU Langone Medical Center, New York) sites of the Autism Brain Imaging Data Exchange II (ABIDE-II) (Di Martino et al., 2017)). These sites were the only ones that included participants in the PREBO-6 age range (~6 years). All scans underwent the same preprocessing and quality-control (QC) pipeline applied to the PREBO-6 data (Methods section 2.3.2).

*HBN dataset*: From the Healthy Brain Network (HBN; releases 3–10) we screened all children aged ≥ 6 y and < 7 y who had resting-state BOLD runs labelled cmrr and acquired on a Siemens 3 T Prisma, identical hardware to PREBO-6. Raw acquisition parameters were: 60 axial slices; 2.4 × 2.4 × 2.4 mm; TR = 800 ms; TE = 30 ms; flip angle = 31°; multiband factor = 6; no phase partial-Fourier. Of 92 eligible participants, 57 had > 6 min of data; after censoring volumes with framewise displacement (FD) > 0.9 mm, 24 were excluded for excessive motion (mean RMS ≥ 0.55 mm) or < 4 min usable data, leaving 33 subjects for analysis.

*Combined external sample*: In total, 40 external term controls were added to the resting-state analysis (7 ABIDE-II, 33 HBN). Median age at scan was 6.6 y (IQR 6.4–6.8). Age distributions by dataset were: ABIDE-II median = 6.4 y (IQR 5.9–6.7) and HBN median = 6.7 y (IQR 6.5–6.9).

## MRI procedures before acquisition and a motion minimisation technique during the scan.

A mock scanning session was conducted to improve participant cooperation, simulating the MRI experience with a hospital bed and MRI-like environment. Participants were familiarised with the MRI protocols through age-appropriate educational materials (Barnea-Goraly et al., 2014; De Bie et al., 2010). This session simulated the MRI experience using a hospital bed, straps, and a tent-like structure simulating the MRI scanner. An age-tailored PowerPoint educated participants on the structural, task-based, and resting-state fMRI protocols. The session also included acoustic examples from gradient coil noises for each sequence to acclimate them to the expected sounds during scanning.

During the scan, to achieve minimal motion during acquisition, we secured the participant's position inside the head coil by using foam pads, placing one over each ear and another on top of the head. Additionally, a researcher (KM) stayed in the scanner room, attentively monitoring the participant's comfort and identifying any significant movements during the scan.

## Functional MRI Analysis

## fMRI Task-Based Data Pre-Processing and First-Level Analysis

Only subjects with fMRI runs containing relative root-mean-squared (RSM) displacement less than half a voxel’s width were included in the analysis (i.e., mean RMS < 1.3) (Power et al., 2012). The off-resonance field of task-based EPI images was estimated using reversed phase-encoding direction pairs and corrected with FSLTOPUP (FSL version 6.0.5) (Smith et al., 2004) followed by standard pre-processing steps as implemented in SPM12 (Wellcome Department of Imaging Neuroscience, UCL, London, UK), including slice-timing correction, realignment to compensate for bulk movements, normalisation to MNI space and spatial smoothing with a Gaussian kernel of 6 mm FWHM. Framewise displacement (FD) measures the amount of head motion from one volume to the next by summing the absolute values of the derivatives of the six realignment parameters (x, y, z translations and rotations). This provides a single metric that summarises the total head movement between consecutive volumes, as described by Power et al. (2012). FD was used to identify and censor volumes with excessive motion, improving fMRI data quality (Ciric et al., 2017; Siegel et al., 2014). Paediatric studies are known for higher head motion, and therefore we set an FD threshold at < 0.9, which balances motion control with statistical power (Siegel et al., 2014). GLM analysis was performed using regressors generated by convolving the time course of the motor task onset and duration with the SPM12 canonical hemodynamic response function with dispersion and temporal derivatives. To account for non-neuronal signal contributions, time courses derived from the 6-rigid motion realignment parameters, white matter, and cerebral spinal fluid (CSF) time courses were also included in the GLM as nuisance regressors (Behzadi et al., 2007). Subject-level activations were determined using family-wise error correction set at 0.05.

## Second-Level Task-Based fMRI Data Analysis

We compared motor network activations from hand-tapping tasks between VPT and TC groups using single-subject parameter estimate maps. Due to participant imbalances, a non-parametric permutation test via SnPM12 (Nichols & Holmes, 2002) was used to avoid assumptions about the data distribution and control Type I error rates (Eklund et al., 2016).

For each hand, motor network activations were identified using a whole-brain one-sample t-test, that was used to guide the motor network localisation and lateralisation analysis. Differences between VPT and TC groups were explored using a two-sample t-test with the contrast [VPT > TC] and [VPT < TC]. These tests, integrated within SnPM analysis, involved 10,000 permutations per fMRI run and a family-wise error correction threshold of p=0.05.

# fMRI Resting-State Data Pre-Processing and Analysis

The resting-state functional data underwent several pre-processing steps. These steps began with the correction of susceptibility-induced off-resonance field of the resting-state EPI images, which was estimated using the pairs of images with reversed phase-encoding directions and corrected using FSLTOPUP, followed by the removal of the first 10 volumes and rigid-body realignment to the mean image, which was performed using the FSL’s MCFLIRT routine from FMRIB Software Library (Smith et al., 2004). As described in Satterthwaite et al. (Satterthwaite et al., 2012), subjects with gross motion > 0.55mm (mean RMS) were excluded from further analysis. Undistorted and realigned resting-state fMRI data set and realignment parameters were pre-processed in CONN-toolbox v.21a (Whitfield-Gabrieli & Nieto-Castanon, 2012), relying upon SPM12 in MATLAB R2021b (The MathWorks Inc, Natick, MA, USA). To minimise the effect of motion, potential outlier frames were identified using ARtifact detection Tools (ART toolbox) (Whitfield-Gabrieli et al., 2011). Frames with FD above 0.9 mm or global BOLD signal changes above five standard deviations were censored, and only subjects with more than 4 minutes of remaining resting-state data post-censoring were included, ensuring sufficient data for reliable connectivity estimates (Parkes et al., 2018; Satterthwaite et al., 2012). Functional and anatomical data were segmented into grey matter, white matter, and CSF tissue classes, normalised into standard MNI space, and resampled to 2 mm isotropic voxels following a direct normalisation procedure (Nieto-Castanon, 2022) using SPM unified segmentation and normalisation algorithm (Ashburner & Friston, 2005). Physiological noise components were estimated using the CompCor method (Behzadi et al., 2007) as implemented in CONN (Nieto-Castanon, 2022). This approach extracted the average BOLD signal and the largest principal components from white matter and CSF within each subject’s eroded segmentation masks, ensuring orthogonality to the global BOLD signal and motion parameters. These components, which capture the time course of physiological noise sources such as cardiac and respiratory fluctuations and CSF pulsations, were regressed from the data alongside motion parameters to minimise non-neural signal contributions.

Functional data were denoised using the CONN standard denoising pipeline (Morfini et al., 2023; Nieto-Castanon, 2020). This included regressing out confounding effects from white matter and CSF, motion parameters and their derivatives, scrubbing regressors, session effects and their derivatives, and linear trends within each run. The BOLD time-series were then bandpass filtered between 0.008Hz and 0.09Hz. Post-denoising, voxel-to-voxel connectivity distributions were visualised for each step. All participants exhibited normally distributed data post-denoising and were included in further analyses. Finally, the data were smoothed using a Gaussian kernel of 6mm FWHM.

### **ROI-to-ROI Functional Connectivity Analysis**

Differences between VPT and TC resting-state functional connectivity were performed using ROI-to-ROI analysis at the cortical and subcortical levels. Connectivity matrices were estimated among 32 seed/target cortical and cerebellar ROIs, as defined in the CONN toolbox (Nieto-Castanon & Whitfield-Gabrieli, 2023). The networks included the Default Mode Network (DMN) comprising the medial prefrontal cortex (MPFC), precuneus cortex (PCC), and bilateral lateral parietal (LP) regions; the Sensorimotor Network (SMN), including superior and bilateral lateral sensorimotor regions; the Visual Network (VIS) encompassing medial, occipital, and bilateral lateral visual regions; the Salience Network (Sal) consisting of the anterior cingulate cortex (ACC), bilateral anterior insula (AI), rostral prefrontal cortex (RPFC), and supramarginal gyrus (SMG); the Dorsal Attention Network (DAN) involving bilateral frontal eye field (FEF) and intraparietal sulcus (IPS); the Frontoparietal Network (FPN) covering the bilateral lateral prefrontal cortex (LPFC) and posterior parietal cortex (PPC); the Language Network (LAN) comprising the bilateral inferior frontal gyrus (IFG) and posterior superior temporal gyrus (pSTG); and the Cerebellar Network (CER) including the anterior and posterior cerebellum. At the subcortical level, ten ROIs from the FSL Harvard-Oxford Atlas maximum likelihood subcortical atlas included in CONN were used. These ROIs encompassed the striatum (right and left caudate, putamen, accumbens, pallidum) and right and left thalamus.

The connectivity between each pair of ROIs was then calculated as the Pearson's bivariate correlation coefficient between their respective time-series. Finally, these correlation coefficients were Fisher's z-transformed, as implemented in CONN (Nieto-Castanon & Whitfield-Gabrieli, 2023), to improve normality for subsequent group-level statistical analyses.

The group-level analyses were performed using a General Linear Model. For each individual voxel, a separate GLM was estimated, with first-level connectivity measures at this voxel as dependent variables and groups (VPT vs TC) as independent variables. Age at scan, sex, handedness (assessed during the MABC-2 at school age), site (different data collection cohorts) and framewise displacement were included as covariates. Voxel-level hypotheses were assessed using multivariate statistics with random effects across subjects and covariance estimation. Inferences were drawn at the cluster level (groups of neighbouring voxels)(Nieto-Castanon, 2020). Cluster-level inferences were based on nonparametric statistics from randomisation/permutation analyses (Bullmore et al., 1999; Nichols et al., 2017), with 1000 residual-randomisation iterations. Results were thresholded using a combination of a cluster-forming p < 0.01 voxel-level threshold and a familywise corrected p-FDR < 0.05 cluster-mass threshold as implemented in CONN.

## Lateralisation Analysis of the BOLD Response

To investigate potential differences in the lateralisation of the BOLD response between VPT and TC, we analysed motor and non-motor regions using the Laterality Index (LI) derived from each individual unthresholded statistical parametric map with the LI-tool (Wilke & Schmithorst, 2006).

The LI-tool calculates the lateralisation of the BOLD response as:

$$LI= \frac{nL-nR}{nL+nR}$$

where nL and nR represent the count of activated voxels in the left (L) and right (R) regions of interest, respectively. A bootstrap approach was used to iteratively calculate 10,000 indices at varied thresholds, producing a robust mean, max, min LI, and confidence interval for each index. These were used to obtain an overall weighted bootstrapped LI per subject.

An absolute LI value of 0.10 was used to determine lateralisation: values above 0.10 indicated left lateralisation, values below -0.10 indicated right lateralisation, and values between -0.10 and 0.10 (inclusive) indicated ambiguous or ‘bilateral’ activation. Lateralisation was assessed at two levels: for lobes showing motor activation (informed by the one-sample *t*-test results) and for lobes not showing motor activation in the second-level analysis. Analyses were performed separately (i) in lobes that exhibited task activation at the second level (frontal, parietal, cerebellum) and (ii) in lobes without activation (e.g., temporal, occipital). Group differences were assessed with two-tailed Mann–Whitney U tests and Bonferroni correction for the number of lobes examined.

| Table S2 – Lateralisation Indices (LI) In Non-Motor Lobes (Temporal, Occipital) During Hand Tapping Task | | | | | |
| --- | --- | --- | --- | --- | --- |
| Lobe | Group | Hand | LI | Lateralisation | Significance (Bonferroni corrected) |
| Temporal | VPT | Right | 0.1 | Left | * 0.049 (Unpaired t-test) |
|  | TC |  | -0.1 | Right |  |
|  | VPT | Left | 0.1 | Left | 0.97 (Unpaired t-test) |
|  | TC |  | 0.1 | Left |  |
| Occipital | VPT | Right | -0.2 | Right | 0.53 (Unpaired t-test) |
|  | TC |  | -0.1 | Right |  |
|  | VPT | Left | 0.1 | Left | 0.19 (Unpaired t-test) |
|  | TC |  | 0.3 | Left |  |
| Lateralisation Indices (LI) of VPT and TC during Right- and Left-Hand Motor Tapping in Brain Lobes without Motor Activation. Lateralisation is determined based on the LI values, with positive values indicating left lateralisation and negative values indicating right lateralisation. Significance values have been Bonferroni corrected. | | | | | |

**Distribution of Lateralisation Indices (LI) Across Brain Lobes During Motor Tasks.**

| **Brain regions with motor activation during motor tapping** | | **Brain regions without motor activation during motor tapping** | |
| --- | --- | --- | --- |
| Right-hand motor task | Left-hand motor task | Right-hand motor task | Left-hand motor task |
| **Frontal** | | **Temporal** | |
|  |  |  |  |
| **Parietal** | | **Occipital** | |
|  |  |  |  |
| **Cerebellum** | |  | |
|  |  |  |  |
| **Fig S2** - Scatter plot of the Lateralisation Indices (LI) for right and left-hand motor tapping (columns) on each respective brain lobe. Each point represents an individual subject, illustrating the distribution and trend of lateralisation within each group. LI was similar for brain lobes showing BOLD activation during tasks. On brain lobes without activation, preterm subjects show contralateral left lateralisation in the temporal lobe during right-hand motor task compared to term control | | | |

*Robustness check: multimodal QC sub-cohort*

Because the right-hand tapping sample (VPT = 43, TC = 15) exceeded the datasets available for left-hand tapping and resting-state analyses, we repeated the LI comparison in the subset whose task (R and L) and resting-state fMRI data all passed QC (n = 34; VPT = 25, TC = 9).

Group comparisons continued to show a significant increase in left temporal lateralisation in VPT children, p = 0.0152, Mann-Whitney test (See figure below). The persistence of the temporal asymmetry in this stringent sub-sample demonstrates that our principal finding is not driven by the larger sample available for the right-hand-only analysis.

**Figure S3 -** Temporal‐lobe laterality indices (LI) during right-hand tapping in the multi-modal QC sub-cohort (n = 34)**.** Dots represent individual participants (blue = term controls, purple = VPT). Horizontal ticks denote the group median; vertical whiskers show the 95 % confidence interval of the median. The dotted vertical line marks LI = 0 (symmetric activation). Negative LI values correspond to left-hemispheric dominance, positive values to right-hemispheric dominance. VPT children exhibited a significantly right-shifted temporal LI relative to controls (Mann–Whitney *U*, *p* = 0.015).

**Table S3 - Brain Regions Showing Significant Activation During Left- and Right-Hand Motor Tasks (Within-Group Analysis).**

| **Motor**  **tapping** | **Group** | **Cluster** | **Brain region** | **MNI coordinates**  **(mm)** | | | **Cluster**  **size** | **Peak**  **T-value** | **Cluster**  **p-value** |
| --- | --- | --- | --- | --- | --- | --- | --- | --- | --- |
|  |  | # | (AAL atlas) | x | y | z | (k) |  | (FWE-corr.) |
| **Left hand > Rest** | VPT  n=36 | 1 | Precentral_R | 34 | -18 | 52 | 1988 | 13.55 | 0.0002 |
|  |  | 2 | Cerebellum_4_5_L | -14 | -52 | -22 | 588 | 12.03 | 0.0002 |
|  |  | 3 | Supp_Motor_Area_R | 8 | -6 | 54 | 302 | 9.65 | 0.0002 |
|  |  | 4 | Rolandic_Oper_R | 44 | -20 | 20 | 134 | 8.57 | 0.0002 |
|  |  | 5 | Thal_VPL_R | 16 | -18 | 6 | 49 | 7.32 | 0.0004 |
|  | TC n=12 | 1 | Cerebellum_6_L | -24 | -48 | -30 | 1964 | 11 | 0.003 |
|  |  | 2 | Precentral_R | 40 | -18 | 56 | 2189 | 10.55 | 0.003 |
| **Right hand > Rest** | VPT  n=43 | 1 | Precentral_L | -38 | -20 | 58 | 2191 | 17.07 | 0.0002 |
|  |  | 2 | Cerebellum_4_5_R | 14 | -50 | -22 | 906 | 15.35 | 0.0002 |
|  |  | 3 | Thal_IL_L | -14 | -20 | 2 | 307 | 12.57 | 0.0002 |
|  |  | 4 | Cerebellum_8_R | 12 | -64 | -48 | 399 | 10.46 | 0.0002 |
|  |  | 5 | Supp_Motor_Area_L | -4 | -4 | 58 | 550 | 9.9 | 0.0002 |
|  |  | 6 | Putamen_L | -32 | -8 | -2 | 213 | 9.04 | 0.0002 |
|  |  | 7 | Rolandic_Oper_L | -48 | 0 | 6 | 55 | 7.73 | 0.0002 |
|  |  | 8 | SupraMarginal_L | -52 | -22 | 18 | 174 | 7.13 | 0.0006 |
|  | TC  n=15 | 1 | Cerebellum_4_5_R | 14 | -50 | -20 | 295 | 11.6 | 0.0004 |
|  |  | 2 | Precentral_L | -32 | -24 | 60 | 696 | 11.03 | 0.0004 |
|  |  | 3 | Thal_VPL_L | -16 | -22 | 4 | 88 | 10.56 | 0.0004 |
|  |  | 4 | Supp_Motor_Area_L | -4 | -4 | 56 | 73 | 8.07 | 0.0014 |
|  |  | 5 | Rolandic_Oper_L | -44 | -22 | 20 | 37 | 7.52 | 0.0038 |
|  |  | 6 | Postcentral_L | -56 | -18 | 50 | 19 | 6.81 | 0.0142 |
|  |  | 7 | Cerebellum_8_R | 12 | -66 | -56 | 16 | 6.79 | 0.0144 |
| The table presents the cluster number, brain region based on the AAL atlas, MNI coordinates, cluster size in voxels (k), peak T-value, and the cluster p-value corrected for family-wise error (FWE voxel-level correction). Regions are identified as follows: Precentral_R/L: Right/Left precentral gyrus. Cerebellum_4_5_L/R: Left/Right cerebellum lobules IV and V. Supp_Motor_Area_R/L: Right/Left supplementary motor area. Rolandic_Oper_R/L: Right/Left rolandic operculum Thal_VPL_R/Thal_IL_L: Right/Left thalamic nuclei. Putamen_L: Left putamen. SupraMarginal_L: Left supramarginal gyrus. Postcentral_L: Left postcentral gyrus. Cerebellum_6_L/8_R: Left/Right cerebellum lobules. | | | | | | | | | |

**Table S4 - Comparison of Motor Task BOLD Activation Metrics Between Very Preterm (VPT) and Term Control (TC) Groups.**

|  | **Right hand motor tapping** | | | | **Left Hand motor tapping** | | |
| --- | --- | --- | --- | --- | --- | --- | --- |
|  | VPT (N=43) | Term (N=15) | Statistic (P) | VPT (N=36) | | Term (N=12) | Statistic (P) |
| 1. **BOLD activation** |  |  |  |  | |  |  |
| M1 Activation Strength (t-value, mean ± SD) | 11.26 (±3.82) | 12.35 (±3.92) | p<0.35 | 12.02 (±3.46) | | 11.74 (±2.15) | p<0.79 |
| Total Activation Volume (cm³) (median, IQR) | 5.3 (2.68-8.58) | 5.86 (3.79-11.22) | p=0.42 | 7.12 (2.45-11.2) | | 5.54 (3.99-11.25) | p=0.99 |
| M1 Activation Volume (cm³) (mean ± SD) | 4.93 (±6.12) | 3.46 (±3.87) | p<0.27 | 5.98 (±5.38) | | 3.86 (±2.72) | p<0.6 |
| **Table S3** – This table presents the BOLD activation metrics, including M1 Activation Strength (t-value, mean ± SD), Total Activation Volume (cm³, median, IQR), and M1 Activation Volume (cm³, mean ± SD). No significant differences were observed in the BOLD activation patterns between the VPT and term-born groups for either hand. | | | | | | | |

|  | **PREBO-6 resting-state** | | | **PREBO-6+ resting-state** | |
| --- | --- | --- | --- | --- | --- |
|  | VPT | TC (PREBO-6) | **Statistic (P)** | TC (PREBO-6+) | **Statistic (P)** |
|  | n=28 | n=12 |  | n=52 |  |
| **Perinatal data** |  |  |  |  |  |
| Gestational Age (weeks) | 28.7 (1.8) | 39.9 (0.7) | *** <2.2e-16 |  |  |
| Birth Weight (g) | 1426.7 (299) | 3414 (294) | *** 6.627e-12 |  |  |
| **Demographic** |  |  |  |  |  |
| Gender (Boys/Girls) | (15/13) | (5/7) | *0.73* | (30/22) | *0.81* |
| Age at MRI (years) (median, IQR) | 6.23 (6.14 - 6.82) | 6.42 (6.2 - 6.6) | *0.57* | 6.56 (6.3 - 6.8) | ******0.0006*** |
| Corrected age at MRI (years) (median, IQR) | 6.1 (6.04 – 6.24) | 6.42 (6.03 - 6.6) | *0.1* | 6.56 (6.3 - 6.8) | ******<0.0001*** |
| Handedness (Right/Left) | (26/2) | (10/2) | *0.57* | (47/5) | *>0.99* |
| **Motion metrics** |  |  |  |  |  |
| RMS | 0.21 (0.16 - 0.29) | 0.3 (0.21 - 0.42) | *0.051* | 0.16 (0.1 - 0.34) | *0.053* |
| FD | 0.21 (0.16 - 0.29) | 0.3 (0.21 - 0.42) | ****0.024*** | 0.22 (0.14 - 0.54) | *0.17* |
| **Table S4 -** Comparison of demographic information, perinatal data, and motion metrics between very preterm (VPT) and term control (TC) groups across two resting-state datasets: PREBO-6 and PREBO-6+. The PREBO-6+ dataset includes additional term controls from the ABIDE-II and HBN datasets. Acronyms: RMS (Root Mean Square), FD (Framewise Displacement). Statistical significance is denoted by (*) where * p<0.05; ** p<0.01; *** p<0.001. | | | | | |

**Table S5 - Participant Characteristics for Resting-State fMRI Analyses (PREBO-6 and PREBO-6+ Cohorts)**

**Supplementary Table S5:** Partial Correlations between Functional Connectivity and Clinical Demographics in the VPT Group

| **Network Type** | **Connection** | **Correlated Variable** | **Spearman's *r*** | **Uncorrected *p*-value** | ***p*-FDR** |
| --- | --- | --- | --- | --- | --- |
| **Cortical Networks** | Salience – Dorsal Attention/Visual | Gestational Age (days) | 0.112 | 0.595 | 0.803 |
|  | Salience – Dorsal Attention/Visual | Birth Weight (g) | 0.402 | **0.04*** | 0.168 |
|  | DMN – FPN | Gestational Age (days) | 0.079 | 0.707 | 0.803 |
|  | DMN – FPN | Birth Weight (g) | 0.051 | 0.803 | 0.803 |
| **Subcortical Networks** | R Caudate – Bilateral Putamen | Gestational Age (days) | 0.054 | 0.798 | 0.798 |
|  | R Caudate – Bilateral Putamen | Birth Weight (g) | -0.245 | 0.227 | 0.303 |
|  | Bilateral Pallidum – L Putamen | Gestational Age (days) | -0.109 | 0.603 | 0.798 |
|  | Bilateral Pallidum – L Putamen | Birth Weight (g) | -0.222 | 0.275 | 0.303 |

Partial correlations between key functional connectivity measures and clinical variables (gestational age and birth weight) within the VPT cohort (n=28), controlling for sex and age at scan. **Cortical Networks:** Weaker connectivity between the salience, dorsal attention, and visual networks showed a nominally significant positive correlation with birth weight (r=0.402, p=0.04), suggesting that lower birth weight is associated with a greater reduction in connectivity between these critical attentional and sensory processing networks. Connectivity between the default mode and frontoparietal networks did not significantly correlate with either clinical variable. No correlations survived correction for multiple comparisons. **Subcortical Networks:** Connectivity within subcortical networks did not show a significant relationship with either gestational age or birth weight.


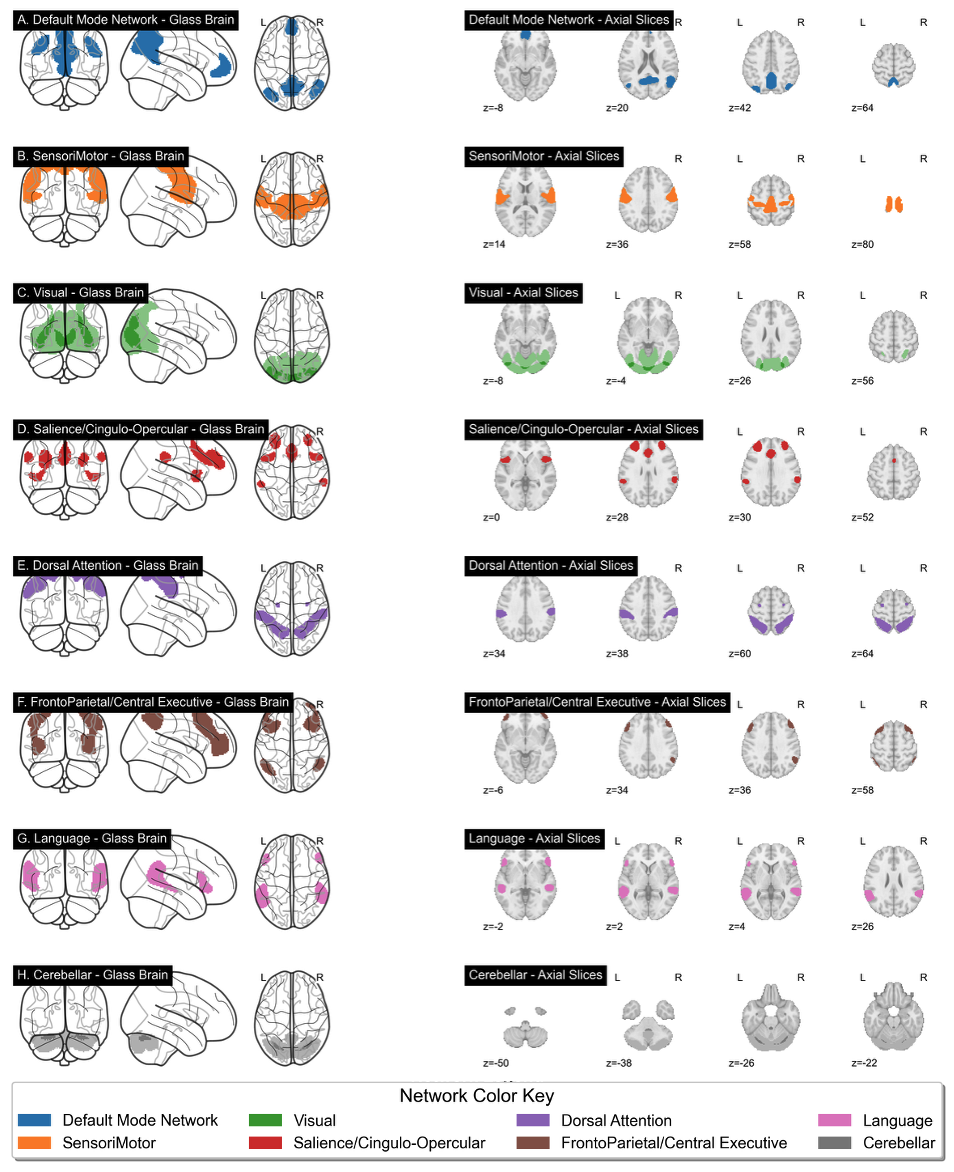


**Figure S4 – CONN Networks Atlas - Eight Canonical Resting-State Networks**

Panels (A-H) depict each network. For each panel, glass brain projections (left) provide multiple spatial views, while axial slices (right) show the network's location at specific z-coordinates . The atlas comprises 32 regions of interest (ROIs) defined from CONN’s Independent Component Analyses of Human Connectome Project dataset (497 subjects) organised into eight canonical resting-state networks: Default Mode Network (4 ROIs), SensoriMotor (3 ROIs), Visual (4 ROIs), Salience/Cingulo-Opercular (7 ROIs), Dorsal Attention (4 ROIs), FrontoParietal/Central Executive (4 ROIs), Language (4 ROIs), and Cerebellar (2 ROIs). Each network is assigned a unique colour, as shown in the key at the bottom legend. Visualisations generated using Nilearn v0.10.4. (Abraham et al., 2014).


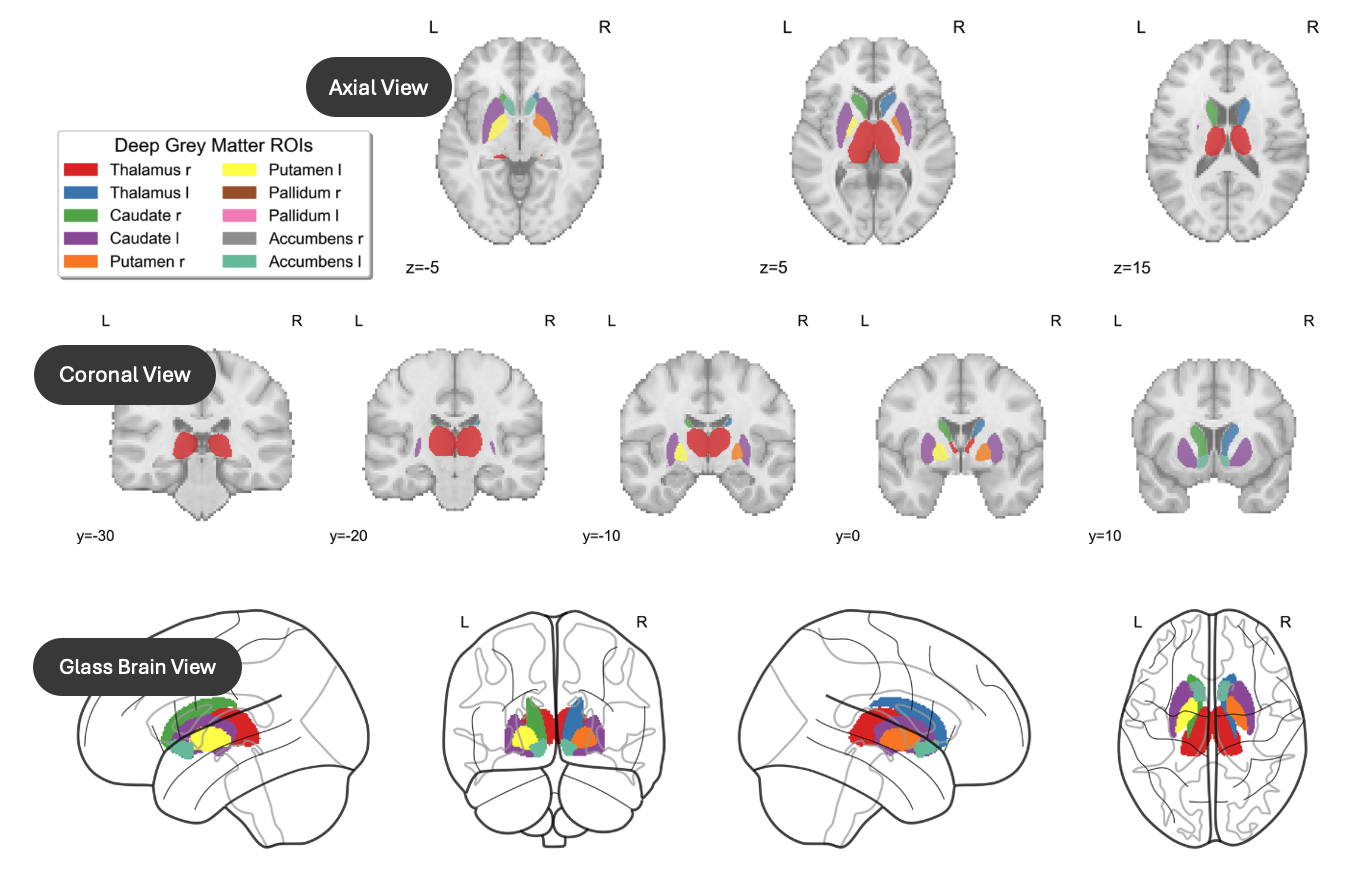


**Figure S5 – Deep Grey Matter ROIs**

Visualisation of 10 bilateral subcortical structures used in the connectivity analysis. ROIs were selected from the FSL Harvard-Oxford maximum likelihood subcortical atlas (HarvardOxford-sub-maxprob-thr25-1mm) within CONN's atlas parcellation. (Top) Axial views at key slice positions showing subcortical structures, (Middle) Coronal views demonstrating bilateral symmetry of the structures, (D) Glass brain projection showing 3D spatial organisation, Color-coded legend (top left) showing each ROI with corresponding labels. Visualisations generated using Nilearn v0.10.4 (Abraham et al., 2014).

# Supplementary Material References:

Abraham, A., Pedregosa, F., Eickenberg, M., Gervais, P., Mueller, A., Kossaifi, J., Gramfort, A., Thirion, B., & Varoquaux, G. (2014). Machine learning for neuroimaging with scikit-learn. *Front Neuroinform*, *8*, 14. <https://doi.org/10.3389/fninf.2014.00014>

Alexander, L. M., Escalera, J., Ai, L., Andreotti, C., Febre, K., Mangone, A., Vega-Potler, N., Langer, N., Alexander, A., Kovacs, M., Litke, S., O'Hagan, B., Andersen, J., Bronstein, B., Bui, A., Bushey, M., Butler, H., Castagna, V., Camacho, N.,…Milham, M. P. (2017). An open resource for transdiagnostic research in pediatric mental health and learning disorders. *Sci Data*, *4*, 170181. <https://doi.org/10.1038/sdata.2017.181>

Ashburner, J., & Friston, K. J. (2005). Unified segmentation. *Neuroimage*, *26*(3), 839-851. <https://www.sciencedirect.com/science/article/pii/S1053811905001102?via%3Dihub>

Barnea-Goraly, N., Weinzimer, S. A., Ruedy, K. J., Mauras, N., Beck, R. W., Marzelli, M. J., Mazaika, P. K., Aye, T., White, N. H., Tsalikian, E., Fox, L., Kollman, C., Cheng, P., Reiss, A. L., & Diabetes Research in Children, N. (2014). High success rates of sedation-free brain MRI scanning in young children using simple subject preparation protocols with and without a commercial mock scanner--the Diabetes Research in Children Network (DirecNet) experience. *Pediatr Radiol*, *44*(2), 181-186. <https://doi.org/10.1007/s00247-013-2798-7>

Behzadi, Y., Restom, K., Liau, J., & Liu, T. T. (2007). A component based noise correction method (CompCor) for BOLD and perfusion based fMRI. *Neuroimage*, *37*(1), 90-101. <https://doi.org/10.1016/j.neuroimage.2007.04.042>

Bullmore, E. T., Suckling, J., Overmeyer, S., Rabe-Hesketh, S., Taylor, E., & Brammer, M. J. (1999). Global, voxel, and cluster tests, by theory and permutation, for a difference between two groups of structural MR images of the brain. *IEEE transactions on medical imaging*, *18*(1), 32-42.

Ciric, R., Wolf, D. H., Power, J. D., Roalf, D. R., Baum, G. L., Ruparel, K., Shinohara, R. T., Elliott, M. A., Eickhoff, S. B., Davatzikos, C., Gur, R. C., Gur, R. E., Bassett, D. S., & Satterthwaite, T. D. (2017). Benchmarking of participant-level confound regression strategies for the control of motion artifact in studies of functional connectivity. *Neuroimage*, *154*, 174-187. <https://doi.org/10.1016/j.neuroimage.2017.03.020>

De Bie, H. M., Boersma, M., Wattjes, M. P., Adriaanse, S., Vermeulen, R. J., Oostrom, K. J., Huisman, J., Veltman, D. J., & Delemarre-Van de Waal, H. A. (2010). Preparing children with a mock scanner training protocol results in high quality structural and functional MRI scans. *European journal of pediatrics*, *169*, 1079-1085.

Di Martino, A., O'Connor, D., Chen, B., Alaerts, K., Anderson, J. S., Assaf, M., Balsters, J. H., Baxter, L., Beggiato, A., Bernaerts, S., Blanken, L. M., Bookheimer, S. Y., Braden, B. B., Byrge, L., Castellanos, F. X., Dapretto, M., Delorme, R., Fair, D. A., Fishman, I.,…Milham, M. P. (2017). Enhancing studies of the connectome in autism using the autism brain imaging data exchange II. *Sci Data*, *4*, 170010. <https://doi.org/10.1038/sdata.2017.10>

Eklund, A., Nichols, T. E., & Knutsson, H. (2016). Cluster failure: Why fMRI inferences for spatial extent have inflated false-positive rates. *Proc Natl Acad Sci U S A*, *113*(28), 7900-7905. <https://doi.org/10.1073/pnas.1602413113>

Morfini, F., Whitfield-Gabrieli, S., & Nieto-Castanon, A. (2023). Functional connectivity MRI quality control procedures in CONN. *Front Neurosci*, *17*, 1092125. <https://doi.org/10.3389/fnins.2023.1092125>

Nichols, T. E., Das, S., Eickhoff, S. B., Evans, A. C., Glatard, T., Hanke, M., Kriegeskorte, N., Milham, M. P., Poldrack, R. A., & Poline, J.-B. (2017). Best practices in data analysis and sharing in neuroimaging using MRI. *Nature neuroscience*, *20*(3), 299-303. <https://www.nature.com/articles/nn.4500.pdf>

Nichols, T. E., & Holmes, A. P. (2002). Nonparametric permutation tests for functional neuroimaging: a primer with examples. *Hum Brain Mapp*, *15*(1), 1-25. <https://doi.org/10.1002/hbm.1058>

Nieto-Castanon, A. (2020). *Handbook of functional connectivity magnetic resonance imaging methods in CONN*. Hilbert Press.

Nieto-Castanon, A. (2022). Preparing fMRI data for statistical analysis. *arXiv preprint arXiv:2210.13564*.

Nieto-Castanon, A., & Whitfield-Gabrieli, S. (2023). *CONN functional connectivity toolbox*. In

Parkes, L., Fulcher, B., Yücel, M., & Fornito, A. (2018). An evaluation of the efficacy, reliability, and sensitivity of motion correction strategies for resting-state functional MRI. *Neuroimage*, *171*, 415-436. <https://doi.org/https://doi.org/10.1016/j.neuroimage.2017.12.073>

Power, J. D., Barnes, K. A., Snyder, A. Z., Schlaggar, B. L., & Petersen, S. E. (2012). Spurious but systematic correlations in functional connectivity MRI networks arise from subject motion. *Neuroimage*, *59*(3), 2142-2154. <https://doi.org/10.1016/j.neuroimage.2011.10.018>

Satterthwaite, T. D., Wolf, D. H., Loughead, J., Ruparel, K., Elliott, M. A., Hakonarson, H., Gur, R. C., & Gur, R. E. (2012). Impact of in-scanner head motion on multiple measures of functional connectivity: relevance for studies of neurodevelopment in youth. *Neuroimage*, *60*(1), 623-632. <https://doi.org/10.1016/j.neuroimage.2011.12.063>

Siegel, J. S., Power, J. D., Dubis, J. W., Vogel, A. C., Church, J. A., Schlaggar, B. L., & Petersen, S. E. (2014). Statistical improvements in functional magnetic resonance imaging analyses produced by censoring high-motion data points. *Hum Brain Mapp*, *35*(5), 1981-1996. <https://doi.org/10.1002/hbm.22307>

Smith, S. M., Jenkinson, M., Woolrich, M. W., Beckmann, C. F., Behrens, T. E., Johansen-Berg, H., Bannister, P. R., De Luca, M., Drobnjak, I., Flitney, D. E., Niazy, R. K., Saunders, J., Vickers, J., Zhang, Y., De Stefano, N., Brady, J. M., & Matthews, P. M. (2004). Advances in functional and structural MR image analysis and implementation as FSL. *Neuroimage*, *23 Suppl 1*, S208-219. <https://doi.org/10.1016/j.neuroimage.2004.07.051>

Whitfield-Gabrieli, S., & Nieto-Castanon, A. (2012). Conn: a functional connectivity toolbox for correlated and anticorrelated brain networks. *Brain Connect*, *2*(3), 125-141. <https://doi.org/10.1089/brain.2012.0073>

Whitfield-Gabrieli, S., Nieto-Castanon, A., & Ghosh, S. (2011). Artifact detection tools (ART). *Cambridge, MA. Release Version*, *7*(19), 11.

Wilke, M., & Schmithorst, V. J. (2006). A combined bootstrap/histogram analysis approach for computing a lateralization index from neuroimaging data. *Neuroimage*, *33*(2), 522-530. <https://doi.org/10.1016/j.neuroimage.2006.07.010>
